# Supplementary material for: Spatial variation and predictors of composite index of HIV/AIDS knowledge, attitude and behaviours among Ethiopian women: A spatial and multilevel analyses of the 2016 Demographic Health Survey
Source: PLoS One. 2024 Jun 4;19(6):e0304982. doi: 10.1371/journal.pone.0304982 (PMC11149886; doi:10.1371/journal.pone.0304982)
Supplement: S1 File — (PDF) [file pone.0304982.s001.pdf]

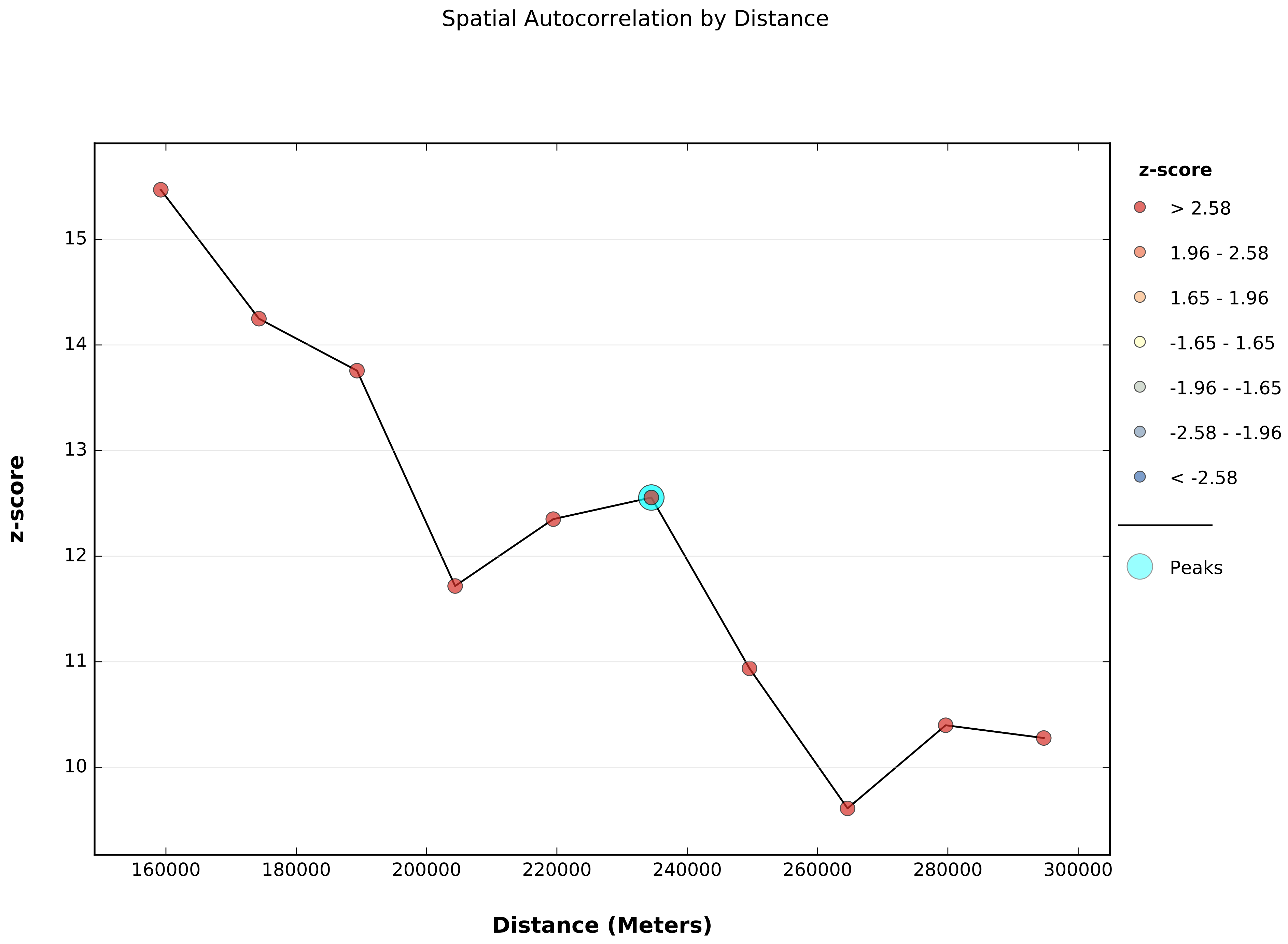

Spatial Autocorrelation by Distance
z-score
Distance (Meters)
15
14
13
12
11
10
160000
180000
200000
220000
240000
260000
280000
300000
z-score
> 2.58
1.96 - 2.58
1.65 - 1.96
-1.65 - 1.65
-1.96 - -1.65
-2.58 - -1.96
< -2.58
Peaks

Global Moran's I Summary by Distance

Distance	Moran's Index	Expected Index	Variance	z-score	p-value
159213.00	0.141681	-0.001634	0.000086	15.470146	0.000000
174269.48	0.120748	-0.001634	0.000074	14.249107	0.000000
189325.97	0.108064	-0.001634	0.000064	13.756656	0.000000
204382.45	0.085789	-0.001634	0.000056	11.717075	0.000000
219438.93	0.080487	-0.001634	0.000044	12.350570	0.000000
234495.42	0.076715	-0.001634	0.000039	12.555419	0.000000
249551.90	0.062081	-0.001634	0.000034	10.937049	0.000000
264608.38	0.050505	-0.001634	0.000029	9.611031	0.000000
279664.87	0.052054	-0.001634	0.000027	10.398531	0.000000
294721.35	0.048927	-0.001634	0.000024	10.277657	0.000000

First Peak (Distance; Value): 234495.42; 12.555419

Max Peak (Distance; Value): 234495.42; 12.555419

Distance measured in Meters

Incremental Autocorrelation Parameters

Parameter Name	Input Value
Input Features	Export_Output_4_Project6
Input Field	P_NO
Number of Distance Bands	10
Beginning Distance	159213.000000
Distance Increment	15056.483246
Distance Method	EUCLIDEAN
Row Standardization	True
Selection Set	False
