# Supplementary material for: Spatial variation and predictors of composite index of HIV/AIDS knowledge, attitude and behaviours among Ethiopian women: A spatial and multilevel analyses of the 2016 Demographic Health Survey
Source: PLoS One. 2024 Jun 4;19(6):e0304982. doi: 10.1371/journal.pone.0304982 (PMC11149886; doi:10.1371/journal.pone.0304982)
Supplement: S2 File — (DOCX) [file pone.0304982.s002.docx]

S1File: The most likely SaTScan clusters of areas with a high prevalence of inadequate knowledge on HIV transmission among women in Ethiopia, EDHS 2016.

| Most likely clusters | Enumeration areas (clusters) identified | Number of clusters | Population | No. of  case | Coordinates / Radius | Relative risk | LLR | P-Value |
| --- | --- | --- | --- | --- | --- | --- | --- | --- |
| 1^st^ most likely cluster | 630, 77, 92, 490, 543, 171, 492, 95, 497, 458, 588, 553, 521, 138, 214, 573, 251, 239, 116, 85, 358, 22, 164, 277, 439, 64, 57, 278, 210, 8, 186, 566, 1, 318, 622, 436, 212, 454, 501, 68, 187, 357, 419 | 43 | 472 | 356 | (7.717178 N, 46.991580 E) / 560.67 km | 1.74 | 96.86 | <0.001 |
| 2^nd^ most likely cluster | 326, 46, 554, 119, 526, 299, 243, 459, 197, 465, 552, 168, 371, 448, 446, 219, 177, 437, 270, 593, 265, 325, 284, 477, 555, 231, 114, 469, 47, 221, 291, 549, 417, 63, 106, 376, 13, 586, 105, 343, 567, 558, 207, 154, 315, 489, 603, 346, 337, 233, 426, 69, 76, 104, 248, 592, 507, 175, 338, 370, 411, 432, 62, 536, 435, 470, 309, 304, 643, 486, 447, 193, 618, 266, 275, 462, 227, 349, 395, 374, 70, 306, 17, 621, 161, 294, 124, 113, 416, 406, 88, 502, 335, 6, 141, 126, 433, 142, 165, 280, 65, 434, 320, 317, 203, 563, 565, 595, 577, 331, 450, 466, 581, 508, 174, 180, 41, 209, 87, 569, 360, 407, 86, 234, 53, 505, 262, 118, 272, 399, 409, 20, 388, 324, 162, 223, 373, 537, 503, 184, 271, 297, 23, 347, 342, 359, 244, 285, 420, 457, 485, 183, 609, 204, 364, 150, 137, 35, 494, 14, 50, 408, 36, 148, 633, 215, 216, 517, 559, 139, 308, 634, 32, 574, 246, 533, 182, 54, 578, 445, 217, 232, 391, 600, 12, 365, 313, 21, 522, 576, 589, 218, 498, 615, 468, 316, 147, 398, 515, 474, 256, 3, 405 | 203 | 3723 | 1945 | (7.528248 N, 35.063386 E) / 426.53 km | 1.27 | 64.52 | <0.001 |
| 3^rd^ most likely cluster | 135, 37, 336, 283, 102, 39, 620, 276, 564, 295, 484, 547 | 12 | 186 | 150 | (10.179467 N, 40.549109 E) / 82.58 km | 1.83 | 51.94 | <0.001 |
| 4^th^ most likely cluster | 599, 544, 488, 344, 249, 348, 332, 241, 128, 130, 442, 427, 172, 389, 511 | 15 | 222 | 166 | (12.569937 N, 40.396640 E) / 86.25 km | 1.69 | 41.88 | <0.001 |
| 5^th^ most likely cluster | 372, 93, 412, 333, 476, 506, 453, 491, 441, 557, 594, 30, 25, 166 | 14 | 207 | 145 | (8.949350 N, 41.312402 E) / 89.61 km | 1.59 | 27.95 | <0.001 |
| 6^th^ most likely cluster | 242, 523, 281, 311 | 4 | 42 | 40 | (9.541209 N, 42.042954 E) / 12.50 km | 2.15 | 25.57 | <0.001 |
| 7^th^ most likely cluster | 134, 263, 192, 117, 362, 103, 127 | 7 | 133 | 95 | (14.179123 N, 39.980749 E) / 39.56 km | 1.61 | 19.89 | <0.001 |
| 8^th^ most likely cluster | 628, 152, 80, 199, 312, 322, 327, 425 | 8 | 149 | 100 | (12.823542 N, 38.243091 E) / 59.91 km | 1.52 | 15.59 | <0.001 |
| 9^th^ most likely cluster | 122, 245, 529 | 3 | 48 | 40 | (8.757437 N, 40.299443 E) / 28.48 km | 1.88 | 15.50 | 0.0017 |
| 10^th^ most likely cluster | 201 | 1 | 21 | 20 | (9.646167 N, 39.617829 E) / 0 km | 2.14 | 12.77 | 0.002 |
