# Supplementary material for: Spatial variation and predictors of composite index of HIV/AIDS knowledge, attitude and behaviours among Ethiopian women: A spatial and multilevel analyses of the 2016 Demographic Health Survey
Source: PLoS One. 2024 Jun 4;19(6):e0304982. doi: 10.1371/journal.pone.0304982 (PMC11149886; doi:10.1371/journal.pone.0304982)
Supplement: Attachment — Submitted filename: comments.pdf [file pone.0304982.s003.pdf]

Thank for inviting to review this interesting paper, the paper has some importance part but it needs substantial improvements

General comment

I would like to emphasize the need for a clear explanation from the authors regarding the rationale behind conducting both multilevel and geographical weighted regression analyses. Specifically, it is essential to address why the geographical weighted regression (GWR) was chosen and how it demonstrates local statistical significance spatially. If GWR already indicates spatial significance, the inclusion of multilevel logistic regression requires justification to avoid redundancy and ensure the efficiency of the analysis. Clarification on this aspect is strongly warranted to strengthen the paper's coherence and contribute to a better understanding of the analytical approach

Specific comments

Abstract

1. The methodology section lacks depth as it fails to mention the specific software utilized for spatial analysis, such as the versions of ArcGIS and SaT Scan employed.

2. The Results section lacks clarification on whether the outcomes represent clustering, identification of hotspot areas, or the results of the SatScan analysis. This ambiguity obscures the interpretation of the findings and their significance. To enhance clarity and provide a comprehensive understanding, it is crucial to explicitly state the nature of the outcomes, particularly regarding clustering, hotspot identification, and the results derived from the SatScan analysis. This clarification will enable readers to better comprehend the implications of the study's findings and their relevance to the research objectives.

3. In conclusion, it is evident that the paper lacks a comprehensive and insightful summary based on the findings presented. The absence of a conclusion section deprives readers of a clear synthesis of the research outcomes and their implications. Therefore, it is imperative to craft a conclusion that effectively encapsulates the key findings, discusses their significance, and provides insights for future research or practical applications

Main documents

Material and Methods

4. The paper overlooks an essential aspect by failing to mention the data source and provide a direct link to the DHS portal site. Including this information is crucial for transparency and reproducibility, as it enables readers to access the original data for validation or further analysis. Therefore, it is imperative to incorporate a clear statement specifying the data source and providing a hyperlink to the DHS portal site, ensuring that readers have easy access to the dataset used in the study. This addition will enhance the paper's credibility and facilitate future research endeavors by enabling others to replicate or build upon the findings.

5. The absence of a map depicting the study area is a notable omission in the paper. Including a map provides essential context for readers to visualize the geographical scope of the study and understand the spatial distribution of the variables under investigation. Incorporating a map showcasing the study area not only enhances the clarity of the research but also enables readers to better interpret the findings in relation to geographic features and boundaries. Therefore, it is recommended to include a detailed map illustrating the study area, its relevant geographical features, and any pertinent spatial data layers. This addition will enrich the paper by providing a visual representation of the research context, thereby enhancing its overall comprehensibility and impact.

6. The author's focus solely on community-level variables limited to region and residence overlooks other potentially significant factors such as community-level educational status, wealth status, media exposure, and others. These variables play crucial roles in shaping community dynamics and could significantly influence the outcomes under investigation. Therefore, it is essential to address this oversight and consider incorporating a broader range of community-level

variables in the analysis. By including additional variables such as educational status, wealth status, and media exposure at the community level, the study can provide a more comprehensive understanding of the factors impacting the outcomes of interest. This expansion of variables will enrich the analysis and enhance the depth of insights derived from the research findings.

7. The absence of incremental autocorrelation analysis in the data analysis section is a notable oversight, particularly concerning autocorrelation analysis. Incremental autocorrelation analysis is crucial for understanding spatial dependence at varying distance thresholds, which can reveal important insights into the clustering patterns within the dataset. Without considering distance thresholds, the autocorrelation analysis may lack precision and potentially overlook significant spatial clustering trends. Therefore, it is imperative to address this deficiency and incorporate incremental autocorrelation analysis into the data analysis methodology

8. The Median Odds Ratio (MOR) in the Results section of the multilevel mixed-effect logistic regression analysis is not mention , especially considering its importance in understanding the variation between areas of highest and lowest risk. MOR provides valuable insights into the heterogeneity between different geographical areas, highlighting the extent to which individual-level characteristics versus contextual factors contribute to the observed variations in risk. Therefore, it is crucial to address this omission and include MOR analysis in the Results section.

9. The discussion section appears to lack depth in its interpreting of the findings, particularly in integrating and comparing results from different analytical approaches such as hotspot analysis, SatScan, and others. Each of these methods provides unique insights into spatial patterns and clusters, and their integration can enrich the understanding of the phenomenon under study. Therefore, it is essential to thoroughly discuss and contrast the findings obtained from each method, providing justification for any discrepancies observed.
