# Supplementary material for: Spatial variation and predictors of composite index of HIV/AIDS knowledge, attitude and behaviours among Ethiopian women: A spatial and multilevel analyses of the 2016 Demographic Health Survey
Source: PLoS One. 2024 Jun 4;19(6):e0304982. doi: 10.1371/journal.pone.0304982 (PMC11149886; doi:10.1371/journal.pone.0304982)
Supplement: Attachment — Submitted filename: PONE-D-24-00224_reviewer commenet.pdf [file pone.0304982.s005.pdf]

PLOS ONE

Spatial variation and predictors of inadequate HIV/AIDS knowledge among Ethiopian Women: A spatial and multilevel analyses of the 2016 Demographic Health Survey

--Manuscript Draft--

**Manuscript Number:**	PONE-D-24-00224
**Article Type:**	Research Article
**Full Title:**	Spatial variation and predictors of inadequate HIV/AIDS knowledge among Ethiopian Women: A spatial and multilevel analyses of the 2016 Demographic Health Survey
**Short Title:**	predictors of inadequate HIV/AIDS knowledge among Ethiopian Women
**Corresponding Author:**	Aklilu Habte, MPH<br>WCU: Wachemo University<br>Hossana, ETHIOPIA
**Keywords:**	HIV/AIDS, Knowledge, Women, Spatial, Predictors, Ethiopia
**Abstract:**	Background<br>Although dissemination of health information is one of the pillars of HIV prevention efforts in Ethiopia, a large segment of women in the country still lack adequate HIV/AIDS knowledge. Some studies on the level of women's knowledge about HIV/AIDS were conducted; however, the majority of them were small-scale and overlooked individual, community-level, or spatial predictors. Hence, this study aimed to estimate the prevalence, geographical variation (Hotspots), spatial predictors, and multilevel correlates of inadequate HIV/AIDS knowledge among Ethiopian women.<br>Methods<br>The study conducted using the 2016 Ethiopian Demographic and Health Survey data, included 12,672 women of reproductive age group (15-49 years). The level of knowledge was assessed using 11 items encompassing HIV/AIDS prevention, transmission, and misconceptions. Spatial autocorrelation (Moran's I) used to determine the spatial variation in inadequate HIV/AIDS knowledge. Multilevel multivariable logistic regression was performed, with the measure of association reported using adjusted odds ratio (aOR) with its corresponding 95% CI.<br>Results<br>The prevalence of inadequate HIV/AIDS knowledge among Ethiopian women was 48.9% (95% CI: 48.1, 49.8), with significant spatial variations observed across regions. Higher inadequate knowledge was observed among women living in the poorest wealth quintile(AOR= 1.63; 95% CI: 1.21, 2.18), rural residents(AOR= 1.62; 95% CI: 1.18, 2.22), having no formal education(AOR= 2.66; 95% CI: 2.04, 3.48), non-autonomous(AOR= 1.71; 95% CI: (1.43, 2.28), never listen to radio(AOR= 1.56; 95% CI: (1.02, 2.39), never watched television(AOR= 1.50; 95% CI: 1.17, 1.92), not having a mobile phone(AOR= 1.45; 95% CI: 1.27, 1.88), and not visiting health facilities(AOR= 1.46; 95% CI: 1.28, 1.72). Relatively poor knowledge was identified in some parts of Somali and the southern parts of Afar.<br>Conclusion<br>About half of women had inadequate HIV/AIDS comprehensive knowledge, with significant spatial variation across regions. Strengthening efforts of providing tailored health education, and information campaigns with an emphasis on women who lack formal education, live in rural areas, and poorest wealth quintile are keys to enhancing knowledge. Local and international collaborations have to endeavour to increase women's autonomy by promoting gender equality and empowering women to access HIV/AIDS health information. The media agencies could prioritise the dissemination of culturally sensitive HIV/AIDS information in the local language to women of reproductive age. The identified small geographical hot spots with relatively poor knowledge of HIV/AIDS should be targeted during resource allocation and interventions.
**Order of Authors:**	Aklilu Habte, MPH in Reproductive Health
	Habtamu Mllie Bizuayehu, PhD in Epidemiology
	Yoseph Haile, MPH in Health service management

Powered by Editorial Manager® and ProduXion Manager® from Aries Systems Corporation

	Daniel Niguse Mamo, MSc in Health Informatics<br>Yordanos Sisay Asegedom, MPH in Epidemiology and Biostatstics
**Additional Information:**	
**Question**	**Response**
**Financial Disclosure**<br><br>Enter a financial disclosure statement that describes the sources of funding for the work included in this submission. Review the submission guidelines for detailed requirements. View published research articles from *PLOS ONE* for specific examples.<br><br>This statement is required for submission and **will appear in the published article** if the submission is accepted. Please make sure it is accurate.<br><br>**Funded studies**<br>Enter a statement with the following details:<br>• Initials of the authors who received each award<br>• Grant numbers awarded to each author<br>• The full name of each funder<br>• URL of each funder website<br>• Did the sponsors or funders play any role in the study design, data collection and analysis, decision to publish, or preparation of the manuscript?<br><br>Did you receive funding for this work?	The author(s) received no specific funding for this work.
**Competing Interests**<br><br>Use the instructions below to enter a competing interest statement for this submission. On behalf of all authors, disclose any competing interests that could be perceived to bias this work—acknowledging all financial support and any other relevant financial or non-financial competing interests.<br><br>This statement is required for submission and **will appear in the published article** if	The authors have declared that no competing interests exist

the submission is accepted. Please make sure it is accurate and that any funding sources listed in your Funding Information later in the submission form are also declared in your Financial Disclosure statement.<br><br>View published research articles from *PLOS ONE* for specific examples.<br><br>**NO authors have competing interests**<br><br>Enter: *The authors have declared that no competing interests exist.*<br><br>**Authors with competing interests**<br><br>Enter competing interest details beginning with this statement:<br><br>*I have read the journal's policy and the authors of this manuscript have the following competing interests: [insert competing interests here]*<br><br>* typeset	
**Ethics Statement**<br><br>Enter an ethics statement for this submission. This statement is required if the study involved:<br><br>• Human participants<br>• Human specimens or tissue<br>• Vertebrate animals or cephalopods<br>• Vertebrate embryos or tissues<br>• Field research<br><br>Write "N/A" if the submission does not require an ethics statement.<br><br>General guidance is provided below. Consult the submission guidelines for detailed instructions. **Make sure that all information entered here is included in the Methods section of the manuscript.**	All methods and processes were carried out per the relevant rules and regulations of the Helsinki Declaration. Following registration, the DHS office issued written permission to utilise the DHS datasets. Furthermore, as this is secondary data, the ethics committee of Wachemo University College of Medicine and Health Sciences declared that no formal ethics approval was required, with a written letter at the reference number (WCU/341/2023).

**Format for specific study types**<br><br>**Human Subject Research (involving human participants and/or tissue)**<br>• Give the name of the institutional review board or ethics committee that approved the study<br>• Include the approval number and/or a statement indicating approval of this research<br>• Indicate the form of consent obtained (written/oral) or the reason that consent was not obtained (e.g. the data were analyzed anonymously)<br><br>**Animal Research (involving vertebrate animals, embryos or tissues)**<br>• Provide the name of the Institutional Animal Care and Use Committee (IACUC) or other relevant ethics board that reviewed the study protocol, and indicate whether they approved this research or granted a formal waiver of ethical approval<br>• Include an approval number if one was obtained<br>• If the study involved *non-human primates*, add *additional details* about animal welfare and steps taken to ameliorate suffering<br>• If anesthesia, euthanasia, or any kind of animal sacrifice is part of the study, include briefly which substances and/or methods were applied<br><br>**Field Research**<br><br>Include the following details if this study involves the collection of plant, animal, or other materials from a natural setting:<br>• Field permit number<br>• Name of the institution or relevant body that granted permission	
**Data Availability**<br><br>Authors are required to make all data underlying the findings described fully available, without restriction, and from the time of publication. PLOS allows rare exceptions to address legal and ethical concerns. See the PLOS Data Policy and FAQ for detailed information.	No - some restrictions will apply

A Data Availability Statement describing where the data can be found is required at submission. Your answers to this question constitute the Data Availability Statement and **will be published in the article**, if accepted.<br><br>**Important:** Stating 'data available on request from the author' is not sufficient. If your data are only available upon request, select 'No' for the first question and explain your exceptional situation in the text box.<br><br>Do the authors confirm that all data underlying the findings described in their manuscript are fully available without restriction?	
**Describe where the data may be found in full sentences. If you are copying our sample text, replace any instances of XXX with the appropriate details.**<br><br>• If the data are **held or will be held in a public repository**, include URLs, accession numbers or DOIs. If this information will only be available after acceptance, indicate this by ticking the box below. For example: *All XXX files are available from the XXX database (accession number(s) XXX, XXX.*).<br>• If the data are all contained **within the manuscript and/or Supporting Information files**, enter the following: *All relevant data are within the manuscript and its Supporting Information files.*<br>• If neither of these applies but you are able to provide **details of access elsewhere**, with or without limitations, please do so. For example:<br><br>*Data cannot be shared publicly because of [XXX]. Data are available from the XXX Institutional Data Access / Ethics Committee (contact via XXX) for researchers who meet the criteria for access to confidential data.*<br><br>*The data underlying the results presented in the study are available from (include the name of the third party*	The data for this study were obtained from the DHS program with a reasonable request. Thus, the one who needs the data supporting the findings of this study can get it in anonymised form from the DHS website at https://dhsprogram.com/Countries/ upon reasonable request in the same manner as the authors did.

and contact information or URL).<br>• This text is appropriate if the data are owned by a third party and authors do not have permission to share the data.<br><br>* typeset	
Additional data availability information:	

Manuscript Click here to access/download;Manuscript;Mnauscript.docx

Spatial variation and predictors of inadequate HIV/AIDS knowledge among Ethiopian Women: A spatial and multilevel analyses of the 2016 Demographic Health Survey

Aklilu Habte[1*], Habtamu Mellie Bizuayehu[2] , Yoseph Haile[3], Daniel Niguse Mamo[4], Yordanos Sisay Asgedom[5]

[1] School of Public Health, College of Medicine and Health Sciences, Wachemo University, Hosanna, Ethiopia

[2] School of Public Health, Faculty of Medicine, the University of Queensland, Brisbane, Australia

[3]Department of public health, school of medicine, college of health science, Arba Minch University, Arba Minch, Ethiopia

[4]Department of Health Informatics, School of Public Health, College of Medicine and Health Sciences, Arba Minch University, Arba Minch, Ethiopia

[5]Department of Epidemiology, College of Health Science and Medicine, Wolaita Sodo University, Wolaita Sodo, Ethiopia

*akliluhabte57@gmail.com

Abstract

Background

Although dissemination of health information is one of the pillars of HIV prevention efforts in Ethiopia, a large segment of women in the country still lack adequate HIV/AIDS knowledge. Some studies on the level of women's knowledge about HIV/AIDS were conducted; however, the majority of them were small-scale and overlooked individual, community-level, or spatial predictors. Hence, this study aimed to estimate the prevalence, geographical variation (Hotspots), spatial predictors, and multilevel correlates of inadequate HIV/AIDS knowledge among Ethiopian women.

Methods

The study conducted using the 2016 Ethiopian Demographic and Health Survey data, included 12,672 women of reproductive age group (15-49 years). The level of knowledge was assessed using 11 items encompassing HIV/AIDS prevention, transmission, and misconceptions. Spatial autocorrelation (Moran's I) used to determine the spatial variation in inadequate HIV/AIDS knowledge. Multilevel multivariable logistic regression was performed, with the measure of association reported using adjusted odds ratio (aOR) with its corresponding 95% CI.

Results

The prevalence of inadequate HIV/AIDS knowledge among Ethiopian women was 48.9% (95% CI: 48.1, 49.8), with significant spatial variations observed across regions. Higher inadequate knowledge was observed among women living in the poorest wealth quintile(AOR= 1.63; 95% CI: 1.21, 2.18), rural residents(AOR= 1.62; 95% CI: 1.18, 2.22), having no formal education(AOR= 2.66; 95% CI: 2.04, 3.48), non-autonomous(AOR= 1.71; 95% CI: (1.43, 2.28), never listen to radio(AOR= 1.56; 95% CI: (1.02, 2.39), never watched television(AOR= 1.50; 95% CI: 1.17, 1.92), not having a mobile phone(AOR= 1.45; 95% CI: 1.27, 1.88), and not visiting health facilities(AOR= 1.46; 95% CI: 1.28, 1.72). Relatively poor knowledge was identified in some parts of Somali and the southern parts of Afar.

Conclusion

About half of women had inadequate HIV/AIDS comprehensive knowledge, with significant spatial variation across regions. Strengthening efforts of providing tailored health education, and information campaigns with an emphasis on women who lack formal education, live in rural areas, and poorest wealth quintile are keys to enhancing knowledge. Local and international collaborations have to endeavour to increase women's autonomy by promoting gender equality and empowering women to access HIV/AIDS health information. The media

agencies could prioritise the dissemination of culturally sensitive HIV/AIDS information in the local language to women of reproductive age. The identified small geographical hot spots with relatively poor knowledge of HIV/AIDS should be targeted during resource allocation and interventions.

Introduction

Human immunodeficiency virus (HIV) and Acquired immunodeficiency syndrome (AIDS) remain a global public health issue, with 40.4 million deaths and 39.0 million individuals living with HIV in 2022; Africa was responsible for two-thirds (25.6 million) of infections worldwide [1]. In Ethiopia, adult (15-49) HIV prevalence was 0.93% in 2019, with higher infections among women compared to men (1.22% vs 0.64%). There were broad geographical variations of HIV infections in Ethiopia, with the highest prevalence in Gambella (4.8%) and Addis Ababa (3.42%) and the lowest in Somalia (0.01%)[2].

Sustainable Development Goal 3 (SDG3) focuses on hindering HIV/AIDS spread, which necessitates not only fostering for equipping the population with evidence-based knowledge about HIV/AIDS but also ensuring equity of information access across various geographic areas [3, 4]. Achieving comprehensive HIV/AIDS knowledge at the population level is among the well-known strategies for HIV/AIDS prevention [5, 6] through reducing HIV-related stigma, and encouraging testing and seeking treatment [7].

New HIV infections among reproductive-aged (15-49 years) women are increasing, which is partly attributed to by lack of comprehensive knowledge about HIV/AIDS transmission and prevention [8, 9]. Thus, to reduce the epidemic, the general public especially women of reproductive age needs to have adequate information regarding HIV transmission, prevention and misconceptions[9-11]. Globally, less than 30% of women of reproductive age have an in-depth knowledge of HIV/AIDS[12, 13]. African women have also limited knowledge regarding how HIV is transmitted, the repercussions of infection, and methods of prevention[14-17].

One of the pillars of HIV prevention efforts in Ethiopia has been the dissemination of information about HIV transmission to enable individuals to identify and avoid engaging in behaviours that raise the risk of HIV transmission[18, 19]. However, a significant proportion of women in the country still lack adequate knowledge about HIV/AIDS[18, 20, 21]. According to the Ethiopian Demographic and Health Survey (EDHS) 2016 report, only 20% of women (15–49 years) have comprehensive knowledge of HIV/AIDS[22]. Socio-demographic and economic factors (age, marital status, literacy, wealth index), region, residence, media exposure, history of HIV test testing and counselling, age at marriage, and

history of multiple sexual encounters were all known to influence the level of comprehensive knowledge[18, 20, 23].

Although some studies[20, 23-25] tried to investigate women's level of comprehensive knowledge, most of them were small-scale studies, mainly focused on knowledge of prevention of mother-to-child transmission, and did not address individual and community-level determinants, as well as spatial predictors. In addition, previous studies have not addressed awareness regarding some misconceptions[20]. Hence, this study aimed to estimate the prevalence, geographical variation (hotspots), spatial predictors, and multilevel (individual and contextual) correlates of inadequate HIV/AIDS comprehensive knowledge (transmission, prevention, and misconceptions) among Ethiopian women.

HIV/AIDS is one of the top priorities of the public health agenda in Ethiopia and there are efforts to enhance knowledge at various administrative units, but there is limited evidence regarding the regions and locations that performed well or limited achievement, which will be addressed by the spatial analysis in this study. The evidence at the finer geographical locations (small administrative units e.g. kebele) will be important to resource prioritisation and tailored interventions[26]. The findings will also help to understand individual and community-level predictors of comprehensive HIV/AIDS knowledge, offering insights that can inform targeted interventions, policy decisions, and community empowerment, and ultimately contribute to HIV/AIDS prevention and equity.

Methods

Data and population

The study was conducted using data sourced from the 2016 Ethiopian Demographic and Health Survey (EDHS), a nationally representative survey[22]. All reproductive-aged (15-49 years) women who participated in EDHS and responded to the knowledge assessment questions were included in this study. Women who have missing data about their residential area locations via geographical positioning system (GPS) were excluded from the spatial analysis.

Sampling procedure and data collection tools

A stratified two-stage cluster sampling technique was used to select study participants; the first stage involved random selection enumeration areas (clusters), followed by selecting a fixed number of 28 households per cluster based on an equal probability systematic selection. A detailed elaboration of the sampling technique is described elsewhere[21, 27]. Each survey cluster's geographic coordinates were obtained using the Global Positioning System (GPS) [21,

28]. Data were collected by using the Woman's Questionnaire, which contains background characteristics, maternal health service, and health system-related characteristics [22].

Measurement of variables of the study

The outcome variable was the level of women's comprehensive HIV/AIDS knowledge, defined as the understanding of modes of transmission, means of prevention, and misconceptions. Knowledge was assessed by 11 questions that dealt with condom utilization, abstinence, modes of transmission, and means of prevention (Table 4). The responses were coded as (1= correct, and 0=incorrect). A composite index that ranges from 1 to 11 was created, and it was then dichotomized into inadequate knowledge (≥ mean score=1) and adequate knowledge (below mean score =0) [29].

Explanatory variables

Following a review of relevant literature [16, 20, 30-32], individual and community-level determinants of comprehensive knowledge about HIV were identified. Individual-level characteristics included socio-demographic, obstetric, and healthcare-related aspects that were unique to each woman, and community-level factors, such as residence and region, were shared by all women residing in the same community (cluster) (Table 1).

Table 1: List of potential predictors of comprehensive knowledge of HIV extracted from the EDHS 2016 report

**Individual-level factors**	
Variables	Description and categorisation
Age	Women's age at the time of the survey and categorised as 15–19, 20–34, and 35-49 years
Marital status	Current status of marriage or cohabitation, categorised as cohabited, never in union, and non-marital relation
Educational status	Categorised as having no formal education, primary, secondary, and higher education
Family size	Number of household members at the time of data collection and dichotomised as ≤5 and >5
Wealth index	Calculated using easy-to-collect data on a household's ownership of selected assets, such as televisions and bicycles; materials used for housing construction; and types of water access and sanitation facilities. Finally, it was categorised into quintiles; poorest, poorer, middle, richer, and richest
Parity	The number of living children the woman had at the time of the survey, and was grouped as nulliparous, primiparous, multiparous, and grand multiparous
Contraceptive uptake	The proportion of women who ever used any contraceptive and categorised as user and non-user
Recent sexual activity	The proportion of women about their recent sexual activity and the responses were categorised as never had sex, not active in the last 4wk (due to postpartum or not), and active in the last 4 weeks

Difficulty in accessing healthcare	Percentage of women who reported that they have serious problems in accessing health care for themselves when they are sick, by type of problem: Getting permission to go for treatment, getting money for treatment, and ease of distance to the health facility. The responses were categorised as ‘Not big problem’ or ‘Big problem’
Covered by health insurance	Percentage of women and men ages 15-49 covered by any health insurance schemes.
Media exposure	Number of women who are exposed to specific media with various frequencies: read a newspaper, watch television, and listen to radio. The responses were categorised as Not at all, less than once a week, and at least once a week
Autonomy in decision-making *	Responses were categorized into low, middle, and high
**Community-level factors**	
Residence	The area where respondents lived and categorised as urban and rural
Region	The geographically delineated area where the woman was resided at the time of the survey. Three categories were created as Small periphery regions (Afar, Somalia, Benishangul, and Gambella), Major central regions (SNNPRs, Tigray, Amhara, and Oromia), and Metropolitans (Addis Ababa, Dire Dawa, and the Harari region)

*Autonomy in decision-making: was assessed by compiling and categorising replies to three questions about who takes the final decision for the family on purchasing, visiting to relatives, and seeking health care. The response categories were (i) woman alone, (ii) woman and husband/partner, (iii) husband/partner alone, (iv) someone else, and (v) others. For each question, responses (i) or (ii) got a score of 1, indicating good decision-making capacity, whereas the remaining responses received a score of 0, indicating weak decision-making capacity. Each of the three components' responses were summed together to yield an overall score ranging from 0 to 3. Finally, a composite score was divided into two distinct groups: low and high for "0 to 2" and "3" scores[33, 34].

Data management and statistical analysis

Statistical software such as STATA version 14, ArcGIS version 10.7, and SaTScan version 9.6 were used to analyse the results of this study. A weighting factor($\frac{\mathbf{v005}}{\mathbf{1000000}}$) was used to ensure the representativeness of the data which results in robust statistical estimations[35]. Descriptive statistics such as frequencies and percentages were computed.

Spatial analyses

To execute the spatial analysis, the weighted proportions of outcome and explanatory variables were computed in STATA and the spatial analysis was computed using ArcGIS version 10.7, and SaTScan version 9.6.

The global spatial autocorrelation was performed using Moran's index to determine whether inadequate HIV/AIDS knowledge among women was dispersed, clustered, or randomly distributed. Moran's, I value was considered statistically significant at $p<0.05$ indicating

geographical HIV/AIDS knowledge variations (clustered)[36, 37]. The spatial interpolation was used to estimate the proportion of inadequate HIV/AIDS knowledge distribution in un-sampled areas, based on observed measurements in the sampled EAs. Although there are different deterministic and geostatistical interpolation methods available, we opted to use the Ordinary Kriging spatial interpolation method since it incorporates spatial autocorrelation and statistically optimises the weight, and have low residual and mean square error [38, 39].

The Spatial Scan Statistical (SaTScan) analysis

Significant hotspots (spatial clusters) with inadequate comprehensive knowledge were estimated using the Bernoulli model from the SaTScan analysis. When using the Bernoulli model, women with inadequate knowledge were classified as cases, otherwise as controls. To determine the number of observed and expected observations inside the window at each point, SaTScan statistics scanned the space progressively. By using the default maximum spatial cluster size of <50% of the population as an upper limit, it was possible to detect both small and large clusters and ignore clusters containing more than the maximum limit[40]. A likelihood ratio (LLR) test statistic with the corresponding p-value was used to identify and rank the clusters in which the observed proportion of inadequate comprehensive knowledge was significantly higher than the expected one or not [41].

Getis-Ord Gi* statistics were performed to identify significant hot spot and cold spot areas for inadequate comprehensive knowledge about HIV transmission and prevention. The Z-score and p-value were determined to figure out the statistical significance of clustering; a high and low Gi* denote a "hot spot," and "cold spot." respectively [42, 43].

Spatial regression

Global (ordinary least squares) and local (geographically weighted regression) techniques are used in spatial regression analysis[44, 45].

Ordinary Least Squares (OLS) regression is a global(model that predicts only one coefficient per independent variable over the whole study area[45]. Assumptions such as the statistical significance of the intercept and predictors, and the presence of multicollinearity among explanatory variables were checked. Variables having multicollinearity (variance inflation factors (VIF>10)) were removed from the model iteratively [44, 46]. Furthermore, The Koenker statistics in the model, revealed a statistically significant p-value (p<0.001), indicating that the global regression model is inconsistent across the study area, suggesting that the local (GWR) model is required to appropriately estimate the model parameters. Finally, a model comparison between global and local regression analysis was done, and the local model

outperformed the global one on the basis of increase in adjusted R2 and decrease in Akaike's Information Criterion (AIC) (Tables 4 and 5).

Multilevel mixed-effect regression model.

We employed multilevel modelling due to the DHS data were hierarchical, and as the outcome variable was binary, we used multilevel multivariable logistic regression analysis.

A multilevel bivariable logistic regression analysis was performed to look into the independent association between knowledge and explanatory variables, with variables with $p< 0.25$ included in multivariable analysis. Statistically significant factors (at $p< 0.05$) along with their adjusted odds ratio(AOR) were reported. At a cutoff point of 10, the Variance Inflation Factor (VIF) was used to detect multicollinearity among covariates and found none (the VIF ranged from 1.01 to 1.97 with a mean of 1.74).

Random effects (measures of variation)

Four separate models were fitted: the first (model 1) was without any covariate (empty model), the second (model 2) merely had individual-level factors, the third (model 3) with only community-level variables, and the fourth (Model 4/full model) with both individual- and community-level variables. For measures of variation, the intra-class correlation coefficient (ICC) and proportional change in variance (PCV) metrics were computed.

ICC is a measure of the degree of heterogeneity of having inadequate comprehensive knowledge between clusters, and it was determined using: $ICC = \frac{\mathrm{var(b)}}{\mathrm{Var(b)+Var(w)}}$, where Var(b) is the variance at the group level and Var(w) is the predicted individual variance component, which is $\pi^2/3 \approx 3.29$.

Proportional Change in Variance (PCV) was estimated as

$PCV = \frac{\mathrm{(Va-Vb)}}{\mathrm{Va}} * 100$, where V_a is the variance of the initial model (null model) and V_b=variance of the subsequent models (models 2, 3, and 4).

Following the comparison, the fourth model with the lowest deviance, Schwarz's Bayesian Information Criterion (BIC), and Akaike's Information Criterion (AIC) values was found to be the best-fit model for this study (Table 6).

Ethical consideration and consent to participate

All methods and processes were carried out per the relevant rules and regulations of the Helsinki Declaration. Following registration, the DHS office issued written permission to utilise the DHS datasets. Furthermore, as this is secondary data, the ethics committee of Wachemo University College of Medicine and Health Sciences declared that no formal ethics approval was required, with a written letter at the reference number (WCU/341/2023).

Results

Background characteristics of the respondents

This study considered a weighted sample of 12,672 women with a mean (±SD) age of 27.77 (±9.11) years (Table 2). The vast majority of respondents (87.8% and 74.8%, respectively) were from large central regions and rural residents. Almost two-thirds (64.0%) of women have cohabited marital status, and one-third (30.4%) were found in the richest wealth quintile. In terms of comprehensive HIV/AIDS knowledge, 58.2% of rural residents, 66.8% of women in the poorest wealth quintile, and 64.3% of women lacking formal education were less knowledgeable. Three out of every ten women (30.8%) were multiparous (had 2-4 living children), and nearly three-quarters (72.8%) were not using contraception. The vast majority of women (82.0%) had higher decision-making autonomy, and more than half (55.5%) had visited health facilities in the last 12 months. Inadequate HIV/AIDS knowledge about was low among women with grand multiparous (62.0%), non-autonomous (70.8%), and never watched television (59.3%) (Table 2).

Table 2: Distribution of characteristics of women: inadequate HIV/AIDS knowledge and bivariable analysis, EDHS 2016.

Variable categories	Total Weighted frequency (%) [N=12,672)	Inadequate HIV/AIDS knowledge (%)	COR[95% CI]	p-value
**Current age**				
15-24	5,074(40.0)	2,325(45.8)	Ref.	
25-34	4,302(34)	2,102(48.9)	1.09(0.94, 1.27)	0.231
35-49	3,296(26.0)	1,780(54.0)	1.41(1.21, 1.64)	<0.001
**Residence**				
Urban	3,195(25.2)	693(21.7)	Ref.	
Rural	9,477(74.8)	5,514(58.2)	6.09(5.06, 7.32)	<0.001
**Regions**				
Major central regions	11,251(87.8)	5,760(51.2)	4.35(3.44, 5.49)	<0.001
Peripheral	436(3.4)	272(62.3)	7.56(5.74, 9.97)	<0.001
Metropolitans	985(7.8)	176(17.9)	Ref.	
**Religion**				
Orthodox	5,972(47.1)	2,423(40.6)	Ref.	
Muslim	3,504(27.7)	2,107(60.1)	1.97(1.63, 2.37)	<0.001
Protestant	2,951(23.3)	1,523(51.6)	1.29(1.04, 1.61)	0.023
Catholic	92(0.7)	57(61.9)	1.82(0.79, 4.21)	0.159
Others	153(1.2))	98(63.5)	2.31(1.21, 4.40)	0.011
**Marital status**				
Unmarried	3,409(26.9)	1,380(40.5)	0.79(0.61, 1.02)	0.075
Cohabited	8,109(64.0)	4,314(53.2)	0.99(0.79, 1.24)	0. 968

Others	1,154(9.1)	513(44.5)	Ref.	
**Educational status**				
No education	5,394(42.6)	3,472(64.3)	4.94(4.05, 6.03)	<0.001
Primary	4,724(37.3)	2,246(47.5)	2.74(2.29, 3.28)	0.011
Secondary and higher	2,555(20.1)	489(19.2)	Ref.	
**Occupational ststus**				
Employed	6,615(52.2)	3,169(52.3)	Ref.	
Unemployed	6,057(47.8)	3,038(45.9)	1.10(0.96, 1.27)	0.313
**Wealth index combined**				
Poorest	1,760(13.9)	1,229(66.8)	5.17(3.98, 6.71)	<0.001
Poorer	2,105(16.6)	1,279(60.8)	3.24(2.53, 4.16)	<0.001
Middle	2,377(18.8)	1,419(59.7)	3.11(2.44, 3.97)	<0.001
Richer	2,574(20.3)	1,305(50.7)	2.13(1.67, 2.71)	<0.001
Richest	3,854(30.4)	976(25.3)	Ref.	
**Family size**				
<=5	6,875(54.2)	3,051(44.4)	Ref.	
>5	5,797(45.8)	3,157(54.5)	1.12(0.88, 1.28)	0.219
**Parity**				
Nulliparous	4,365(34.5)	1,786(40.9)	Ref.	
Primiparous	1,669(13.2)	758(45.4)	1.17(0.97, 1.40)	0.079
Multiparous	3,909(30.8)	1,971(50.4)	1.24(1.07, 1.43)	0.004
Grand multiparous	2,730(21.5)	1,693(62.0)	1.61(1.32, 1.95)	<0.001
**Contraceptive usage**				
Yes	3,445(27.2)	1,495(43.4)	Ref.	
No	9,227(72.8)	4,713(51.1)	1.19(1.06, 1.35)	0.123
**Autonomy in decision-making**				
Low	1,243(9.8)	881(70.8)	2.03(1.62, 2.55)	0.002
Middle	1,042(8.2)	587(56.3)	1.34(1.08, 1.66)	0.04
High	10,387(82.0)	4,740(45.6)	Ref.	
**Ease of distance to seek medical care**				
Big problem	5,973(47.1)	3,614(60.5)	1.39(1.18, 1.64)	<0.001
Not a big problem	6,699(52.9)	2,593(38.7)	Ref.	
**Ease of money to seek medical care**				
Big problem	6,527(51.5)	3,791(58.1)	1.53(1.32, 1.78)	<0.001
Not a big problem	6,145(48.5)	2,416(39.3)	Ref.	
**Listen to radio**				
Not at all	8,049(63.5)	4,555(56.6)	2.35(1.94, 2.84)	<0.001
Less than once a week	2,317(18.3)	941(40.6)	1.51(1.21, 1.87)	<0.001
At least once a week	2,305(18.2)	712(30.9)	Ref.	
**Watching TV**				
Not at all	8,627(68.1)	5,113(59.3)	3.96(3.19, 4.91)	<0.001
Less than once a week	1,690(13.3)	651(38.5)	2.11(1.69, 2.63)	<0.001
At least once a week	2,355(18.6)	443(18.8)	Ref.	
**Reading newspaper**				
Not at all	10,663(84.2)	5,776(54.2)	3.09(2.10, 4.57)	<0.001

Less than once a week	1,438(11.3)	298(20.7)	1.04(0.69, 1.55)	0.844
At least once a week	571(4.5)	134(23.4)	Ref.	
**Own mobile phone**				
No	8,753(69.1)	5,193(59.3)	2.93(2.50, 3.52)	<0.001
Yes	3,919(30.9)	1,015(25.9)	Ref.	
**Covered by Health Insurance**				
Yes	716(5.7)	304(42.5)	Ref.	
No	11,956(94.3)	5,903(49.4)	1.11(0.85, 1.45)	0.443
**Visit health facility within the last 1 year**				
Yes	5,634(55.5)	2,342(41.6)	Ref.	
No	7,036(44.5)	3,866(54.9)	1.52(1.33, 1.74)	<0.001

Ref.: Reference, COR: Crude Odds Ratio

The level of inadequate HIV/AIDS knowledge and regional disparities

About half of women (48.9%: 95% CI: 48.1, 49.8) were found to be not knowledgeable. The highest and the lowest proportion of women with inadequate knowledge were reported in Somali (75.4%) and Addis Ababa (15.2%) respectively (Figure 1). Most women have awareness about the transmission of HIV during breastfeeding (91.7%), pregnancy (80.6%), and delivery (86.8%) (Table 3).

Table 3: The level of HIV/AIDS among Ethiopia women, EDHS 2016

**Knowledge assessment**	Yes (%)	No (%)
The use of condoms to reduce HIV transmission	8,329(65.7)	4,343(34.7)
The use of Abstinence to reduce HIV transmission	9,701(76.5)	2,971(23.45)
Getting HIV by mosquito bite	5,830(46.0)	6,843(54.0)
People can get HIV by sharing food with a person who has AIDS	2,414(19.0)	10,258(81.0)
A healthy-looking person can have and transmit HIV	8,619(68.0)	4,054(32.0)
HIV transmitted during pregnancy	10,208(80.6)	2,464(19.4)
HIV transmitted during delivery	11,001(86.8)	1,671(13.2)
HIV transmitted during breastfeeding	11,619(91.7)	1,054(8.3)
Know the place to get an HIV test	9,776(77.2)	2,896(22.8)
Can get HIV by witchcraft or supernatural means	2,415(19.0)	10,258(81.0)
Drugs to avoid HIV transmission to the baby during pregnancy	8,031(63.4)	4,642(36.6)

Fig 1. Regional disparity in the proportion of women with inadequate HIV/AIDS knowledge, EDHS 2016

Results of spatial analysis

Spatial distribution of inadequate knowledge

Somalia, the western borders of Afar, Benishangul Gumuz, and Gambella had a higher proportion of women with inadequate HIV/AIDS knowledge. In contrast, Tigray, Addis Ababa, and the eastern border of Amhara showed good knowledge (Figure 2).

Fig 2. Spatial distribution of women with inadequate HIV/AIDS knowledge, EDHS 2016

Spatial autocorrelation of inadequate knowledge

The global spatial autocorrelation analysis demonstrated that the geographical distribution of inadequate knowledge across the country was non-random (i.e. there was significant spatial variation)(global Moran's I = 0.64, p<0.001). The clustered patterns (on the right sides) suggest that there was a high proportion of inadequate knowledge throughout the study area (Figure 3).

Fig 3. The global spatial autocorrelation of inadequate HIV/AIDS knowledge, EDHS 2016

Hot Spot (Getis-Ord Gi*) analysis

As per hot spot analysis, Somali, and most parts of Afar regions were identified as hot spots for women with inadequate HIV/AIDS knowledge. The central part of Addis Ababa, as well as several parts of the Oromia region, on the other hand, were cold spots (Figure 4).

Fig 4. Hot spot and Cold spot of inadequate HIV/AIDS knowledge, EDHS 2016

Spatial interpolation

The spatial distribution of inadequate HIV/AIDS knowledge for locations where data were not obtained has been estimated using the standard Kriging technique. Accordingly, the highest estimated prevalence of inadequate knowledge (red shaded) was observed in southern Somalia, North and South Afar, and parts of Dire Dawa (Figure 5).

Fig 5. Ordinary Kriging interpolation of the spatial distribution of inadequate HIV/AIDS knowledge, EDHS 2016.

Spatial scan statistical (SaTScan) analysis

The SaTScan geographical analysis showed ten statistically significant groups of SaTScan clusters with a high proportion of women with inadequate knowledge; the prevalence of insufficient knowledge was higher inside the SaTScan circular window than outside one. Furthermore, the analysis came across 306 significant clusters that accounted for inadequate knowledge. The first most likely cluster was located at geographical coordinates (7.717178 N, 46.991580 E) with a 560.67 Km radius, LLR of 96.86, and Relative risk (RR=1.74); women in the area were 74% more likely to be not knowledgeable than their counterparts outside the area. **(S1File)**.

Results of Spatial regression

The global ordinary least square (OLS) analysis results

The OLS model is the initial step towards selecting relevant predictors for the geographical variability of inadequate HIV/AIDS knowledge. Accordingly, inadequate knowledge was found to be associated with a lack of formal education, living in the poorest wealth quintile, a

lack of autonomy in decision-making, and never listening to the radio and watching television. There was no evidence of multicollinearity among the predictors (minimum and maximum VIF of 1.14 and 2.05, respectively). Furthermore, the null model explained 63.1% of the variation in inadequate knowledge (adjusted R2 = 0.631). Jarque-Bera statistics with p-values greater than 0.05 (p=0.118) indicate that the model prediction was not skewed. The Koenker statistics in the model, on the other hand, revealed a statistically significant p-value (p<0.001), indicating that the regression model is inconsistent across the study area, suggesting that the local (GWR) model is required to appropriately estimate the model parameters (Table 4).

Table 4: Summary of OLS results for inadequate HIV/AIDS knowledge, EDHS 2016

Variable	Coefficient	SE	t-Statistic	Probability	Robust SE	Robust t-statistics	Robust probability	VIF
Intercept	1.013	0.024	42.41	0.001	0.023	43.30	<0.001	---
Women without formal education	-0.277	0.036	-7.55	<0.001	0.043	-6.44	<0.001	2.05
Women living in the poorest wealth quintile	-0.083	0.027	-3.06	0.002	0.030	-2.72	0.006	1.80
Non-autonomous women	-0.211	0.053	-3.94	<0.001	0.058	-3.62	0.0003	1.28
Women who never listen to radio	-0.109	0.038	-2.84	0.005	0.041	-2.63	0.009	1.75
Women who never watch television	-0.153	0.033	-4.64	<0.001	0.034	-4.40	<0.001	1.72
Women who didn't visit a health facility within the last 12 months	-0.249	0.036	-6.81	<0.001	0.047	-5.28	<0.001	1.14

OLS Diagnostics

Number of Observations:	613	Akaike's Information Criterion (AICc)	-443.03
Multiple R-Squared	0.636	Adjusted R-Squared [d]:	0.631
Joint F-Statistic	131.89	Prob(>F), (7,599) degrees of freedom	<0.001
Joint Wald Statistic	2118.68	Prob(>chi-squared),(7) degrees of freedom	<0.001
Koenker (BP) Statistic	30.61	Prob(>chi-squared), (7) degrees of freedom	<0.001
Jarque-Bera Statistic	123.21	Prob(>chi-squared), (2) degrees of freedom	0.118

Geographically weighted regression (GWR) analysis

The GWR analysis outperformed the global model (OLS) significantly. The AICc value of -443.03 in the OLS model dropped to -540.27 in the GWR, while the adjusted R2 value of 0.631 in the OLS rose to 0.693 in the GW; demonstrating the necessity of moving from global (OLS) to local (GWR) regression to identify spatial predictors of inadequate knowledge (Tables 4 and 5).

Table 5: Summary of GWR model fitted with potential spatial predictors of inadequate HIV/AIDS knowledge, EDHS 2016.

List of variables	Women without formal education, living in the poorest wealth quintile, non-autonomous in decision making, who never listen to radio, who never watch television, and who didn't visit a health facility within the last 12 months
Residual squares	13.044
Effective number	49.567
Sigma	0.152
AICc	-540.27
Multiple R-Squared	0.718
Adjusted R-Squared	0.693

The GWR model was highly explained (with the highest range of R-square) in Tigray, central Afar, northern parts of Somali, central parts of Dire Dawa and Harari, and eastern borders of Amhara and Addis Ababa (Figure 6).

Fig 6. The spatial mapping of local adjusted R-square of the GWR analysis for selected spatial predictors of inadequate HIV/AIDS knowledge, EDHS 2016

In most parts of Somali, Benishangul Gumuz, and Gambella regions, and the eastern parts of SNNPR, as the proportion of women without formal education rose, so did the proportion of women with inadequate knowledge. On the other hand, in most portions of the Amhara, Tigray, and Afar regions, the association between lack of formal education and inadequate comprehensive knowledge about HIV was found to be relatively weaker (Figure 7).

Fig 7. GWR coefficients of the proportion of women lacking formal education for predicting inadequate HIV/AIDS knowledge, EDHS 2016

This study also highlights the space-dependent relationship between inadequate HIV/AIDS knowledge and the wealth index. As the proportion of women living in the poorest wealth quintile increases, the likelihood of having inadequate knowledge also increases in Somali, Benishangul Gumuz, and vast areas of SNNPR (Figure 8).

Fig 8. GWR coefficients of the proportion of women living in the poorest wealth quintile for predicting inadequate HIV/AIDS knowledge, EDHS 2016

Lack of autonomy in decision-making had a strong link with inadequate knowledge. As the proportion of non-autonomous women rose, so did the level of inadequate knowledge on HIV in Somali followed by Afar, Benishangul Gumuz, and north of the SNNPR (Figure 9).

Fig 9. GWR coefficients of the proportion of non-autonomous women for predicting the level of inadequate HIV/AIDS knowledge, EDHS 2016

As the proportion of women who never listened to the radio increased, the proportion of inadequate HIV/AIDS knowledge in various parts of SNNPR, southwest Somali, the southern

part of Benishangul Gumuz, and northern parts of Gambella. The red-colored clustered points indicate areas where the coefficients were largest, which in turn indicates the strong positive association between the two variables (Figure 10).

Fig 10. GWR coefficients of the proportion of women who never listen to the radio for predicting the level of inadequate HIV/AIDS knowledge, EDHS 2016

Similarly, as the proportion of women who never watched TV increased, the proportion of inadequate knowledge about HIV in various parts of Somali, Dire Dawa, Harari, the western borders of Oromia, and the central part of Benishangul Gumuz (Figure 11).

Fig 11. GWR coefficients of the proportion of women who never watch TV for predicting the level of inadequate HIV/AIDS knowledge, EDHS 2016

As the number of women who did not visit health facilities in the previous 12 months increased, so did the proportion of people with poor knowledge about HIV in most areas of Somali region (Figure 12).

Fig 12. GWR coefficients of the proportion of women who didn't visit health facilities within the last 12 months for predicting the level of inadequate HIV/AIDS knowledge, EDHS 2016

Results of multilevel mixed-effect logistic regression

Random effect (measures of variation)

The ICC in the null model revealed that 33.1% of the variability in inadequate knowledge was explained by between-cluster variability (ICC = 0.331, p<0.001). Furthermore, a proportionate change in variance (PCV) of 81.25% in the full model suggests that individual and community-level factors together accounted for 81.25% of the variability seen in the null model. As we moved from model 1 (the empty model) to model 4 (the full model), the values of AIC, BIC, and Deviance declined, indicating that the final model fitted throughout the study had appropriate goodness of fit. Finally, the fourth model with the lowest deviance (13307.2) was selected as the best model fit for the study (Table 6).

Table 6: Random intercept variances and model fit statistics comparison of multilevel mixed effect logistic regression model

Measures	Model I (null model)	Model II (individual-level factors)	Model III (community-level factors)	Model-IV (full model)
**Random effects**				
Variance	1.60	0.33	0.71	0.30
ICC	0.331	0.091	0.177	0.088
AIC	14952.23	13584.6	14578.1	13573.1
BIC	14967.1	13807.3	14615.2	13818.0

PCV	Ref.	79.3%	55.6%	81.25%
**Model fitness**				
Log-likelihood	-7474.1	-6762.3	-7284.0	-6653.6
Deviance	14948.2	13524.6	14568.0	13307.2

Fixed effects: Predictors of inadequate knowledge about HIV transmission

In the multilevel multivariable logistic regression analysis, wealth index, educational status, autonomy in decision-making, media exposure, mobile ownership, visit to health facilities, residence, and region were identified as significant determinants of the level of comprehensive knowledge of HIV transmission (Table 7).

Women from rural areas had 90% times higher inadequate comprehensive knowledge of HIV (AOR= 1.87; 95% CI: 1.48, 3.85) than their urban counterparts. Similarly, the likelihood of having inadequate knowledge was 62% higher among rural women than among urban women (AOR= 1.62; 95% CI: 1.18, 2.22). Women with no formal education were 2.66 (AOR= 2.66; 95% CI: 2.04, 3.48) times more likely to have inadequate HIV/AIDS knowledge than women with secondary education or higher educational status. The odds of inadequate knowledge were 1.56 (AOR= 1.56; 95% CI: (1.02, 2.39) and 1.50 (AOR= 1.50; 95% CI: 1.17, 1.92) higher among women who never listen to the radio and watched television, respectively. Non-autonomous women had 71% (AOR= 1.71; 95% CI: (1.43, 2.28) higher likelihood of having inadequate knowledge as compared to autonomous women (Table 7).

Table 7: Results of a multilevel multivariable logistic regression to identify the determinants of inadequate knowledge of women on HIV transmission in Ethiopia, EDHS 2016.

Variable categories	Model I (null model)	Model II (individual-level factors)	Model III (community-level factors)	Model-IV (full model)
		AOR(95%CI)	AOR (95%CI)	AOR (95%CI)
**Current age**				
15-24		Ref.		Ref.
25-34		0.90(0.74, 1.09)		0.92(0.75, 1.11)
35-49	`	0.92(0.72, 1.15)		0.95(0.75, 1.19)
**Religion**				
Orthodox		Ref.		Ref.
Muslim		1.62(1.34, 1.94)		**1.54(1.27, 1.87)**
Protestant		1.17(0.96, 1.44)		1.15(0.94, 1.42)
Catholic		2.11(0.77, 5.81)		2.05(0.76, 5.53)
Others		1.63(0.88, 3.01)		1.60(0.86, 2.94)
**Wealth index combined**				
Poorest		1.93(1.49, 2.50)		1.63(1.21, 2.18)
Poorer		1.37(1.08, 1.73)		1.17(0.88, 1.55)

Middle		1.50(1.17, 1.92)		1.29(0.97, 1.73)
Richer		1.25(0.99, 1.57)		1.08(0.82, 1.42)
Richest		Ref.		Ref.
**Educational status**				
No education		2.71(2.08, 3.55)		2.66(2.04, 3.48)
Primary		1.66(1.35, 2.04)		1.64(1.34, 2.02)
Secondary and higher		Ref.		Ref.
**Parity**				
Nulliparous		Ref.		Ref.
Primiparous		1.08(0.87, 1.33)		1.07(0.86, 1.32)
Multiparous		0.84(0.67, 1.05)		0.83(0.66, 1.04)
Grand multiparous		0.91(0.68, 1.21)		0.87(0.65, 1.17)
**Contraceptive usage**				
Yes		Ref.		Ref.
No		1.24(1.08, 1.43)		1.23(0.97, 1.42)
**Autonomy in decision making**				
Low		1.83(1.45, 2.30)		**1.71(1.43, 2.28)**
Medium		1.31(1.04, 1.64)		1.30(0.99, 1.63)
High		Ref.		Ref.
**Ease of distance to seek healthcare**				
Big problem		1.09(0.92, 1.30)		1.07(0.90, 1.28)
Not a big problem		Ref.		Ref.
**Ease of money to seek healthcare**				
Big problem		1.16(0.98, 1.36)		1.07(0.98, 1.38)
Not a big problem		Ref.		Ref.
**Listen to radio**				
Not at all		1.55(1.02, 2.38)		**1.56(1.02, 2.39)**
Less than once a week		0.98(0.65, 1.48)		0.99(0.66, 1.49)
At least once a week		Ref.		Ref.
**Watching TV**				
Not at all		1.63(1.29, 2.05)		**1.50(1.17, 1.92)**
Less than once a week		1.31(1.01, 1.70)		1.23(0.94, 1.615)
At least once a week		Ref.		Ref.
**Reading newspaper**				
Not at all		1.28(1.05, 1.56)		1.19(0.98, 1.58)
Less than once a week		1.21(0.97, 1.53)		1.22(0.97, 1.54)
At least once a week		Ref.		Ref.
**Own mobile phone**				
No		1.56(1.28, 1.89)		**1.45(1.27, 1.88)**
Yes		Ref.		Ref.
**Visit health facility within the last 12 months**				
Yes		Ref.		Ref.
No		1.49(1.29, 1.73)		**1.46(1.28, 1.72)**
**Residence**				

Urban			Ref.	Ref.
Rural			5.71(4.54, 7.18)	**1.62(1.18, 2.22)**
**Regions**				
Major central regions			1.14(0.88, 1.48)	0.97(0.71, 1.15)
Peripheral			2.46(1.87, 3.24)	**1.87(1.48, 3.85)**
Metropolitans			Ref.	Ref.

Key: Ref.: Reference category; aOR=Adjusted Odds Ratio, * statistically significant at p <0.05, ** statistically significant at p <0.001

Discussion

This study assessed the magnitude, spatial variation, and determinants of inadequate HIV/AIDS comprehensive knowledge among Ethiopian women. Nearly half (48.9%), of women were not knowledgeable. There was significant spatial variation in the prevalence of comprehensive knowledge across small geographical areas. The likelihood of having inadequate knowledge was higher among women living in the poorest wealth quintile, residing in rural areas, with no formal education, who were non-autonomous in decision-making, not exposed to media (radio and television), who didn't have mobile and didn't visit health facilities.

The level of inadequate comprehensive HIV/AIDS knowledge in this study was consistent with a study in Uganda (48.1%)[47], but higher than studies conducted in Indonesia (46.4%)[48], China (19.1%)[49], Tajikistan (12.95%)[50], and Pakistan (32%)[29] and lower than findings in Kenya (53.7%) [51], India (74.2%)[52], Iraq (91.2%) [53], Malawi (57.8%) [54] or low and middle-income countries (70.7%) [9] and Sub-Shahran Africa(61.44%) [16]. Disparities in socioeconomic status, educational opportunities and attainment, cultural and societal attitudes towards sex education and HIV/AIDS discussions, gender disparities, access to healthcare facilities and the quality of health services, and the availability, accessibility, and dissemination of accurate HIV/AIDS information could all contribute to the disparities[9, 55, 56]. Thus, the Ethiopian government and stakeholders in the health sector need to work jointly to reduce the high prevalence of inadequate comprehensive HIV/AIDS knowledge through a multifaceted approach: public health campaigns, education programmes, and policy initiatives that can play a significant role in promoting accurate information and awareness.

The study pointed to the need for special emphasis during resource allocation and targeted interventions in Somali and the southern parts of Afar, which showed relatively higher inadequate comprehensive HIV/AIDS knowledge (significant spatial clusters [Hot Spots]). The interventions could be strengthening tailored educational campaigns, community engagement initiatives including support groups, media infrastructure and coverage in the local language. Incorporating culturally sensitive educational materials and disseminating them through local

community leaders and influencers and mobile health initiatives, including SMS campaigns can be employed to ensure widespread access to accurate information. Strengthening healthcare infrastructure in these regions is essential, enhancing accessibility and providing training for healthcare professionals. Higher inadequate comprehensive HIV/AIDS knowledge in Somali and the southern parts of Afar could be attributed to cultural factors, limited access to healthcare facilities and services, limited possibilities for women's empowerment, and the way of life (pastoral communities), impeding the dissemination of health information. Ongoing research and monitoring efforts should be conducted to understand unique drivers for low knowledge and solutions to address them.

Having no formal education was identified as both an individual- and spatial predictor of inadequate comprehensive HIV/AIDS knowledge, which is supported by previous studies[16, 20, 29, 47, 57]. This could be because women who have no formal education may have limited access to HIV/AIDS information, hampering their ability to obtain correct and up-to-date information. In addition, they may lack empowerment, hindering their ability to seek information autonomously, hesitant to seek information or services due to fear of judgment and discrimination, and restricted mobility might lead to a lack of access to essential health information [29, 58]. Thus, a concerted effort is required to enhance access to education and emphasise the provision of HIV/AIDS education tailored to this population group.

In tandem with studies conducted in SSA countries[16], Pakistan[29], Malawi[54], and Uganda[47] inadequate knowledge was higher among women living in the poorest wealth quintile. The possible justifications could be women in the poorest wealth quintile may have limited access to media and healthcare services, including HIV testing and counselling [59]. In addition, the financial challenges can lead to survival-focused priorities, leaving little room for individuals to prioritize health education and could result in inadequate knowledge. Thus, tailored interventions that consider the specific challenges faced by women in the poorest wealth quintile can contribute to improving comprehensive knowledge and reducing the spread of the virus in these communities.

Women’s autonomy in decision-making in accessing information and healthcare has significant implications, particularly for knowledge about HIV/AIDS, a disease that disproportionately affects women. In our study, lacking autonomy in decision-making was found to increase the odds of inadequate HIV/AIDS comprehensive knowledge. Studies conducted elsewhere [20, 29] supported the finding. This could be due to the lack of autonomy could result in economic dependence on male partners or family members, distorted power dynamics, and limited access to health information, which may deter women from seeking

HIV/AIDS information. Thus, increasing gender equality, expanding access to education and healthcare, confronting cultural stigmas, and empowering women to make informed health decisions should be the necessary steps by the government to increase women's autonomy.

In line with studies conducted in SSA countries[16], India [52], and Pakistan[29], lack of media exposure(Radio, Television, and mobile phone) was associated with inadequate comprehensive HIV/AIDS knowledge. This might be due to women who lack media exposure might face limited access to health information and updates regarding modes of transmission and prevention which can hinder the acquisition of comprehensive knowledge about HIV/AIDS. In addition, mobile phones allow women to stay connected and communicate with family, friends, and support networks both in voice conversation and social media platforms which can provide them with a variety of information regarding HIV/AIDS.

Finally, the likelihood of inadequate comprehensive HIV/AIDS knowledge was higher among rural women, which was supported by similar studies [16, 29, 47, 57]. This might be due to rural women having economic challenges, and limited access to educational resources, healthcare facilities, and information campaigns about HIV/AIDS compared to urban areas, which can hinder the dissemination of accurate information about prevention, transmission, and misconceptions. In addition, they might have limited decision-making power or autonomy, which can impact their ability to access education and healthcare. As a result, efforts should be made to improve HIV/AIDS knowledge by emphasising culturally relevant education, widening access to healthcare facilities, empowering women via education and community participation, and tackling socioeconomic inequities.

This study had several strengths. First, the findings were based on an analysis of nationally representative data, thereby enhancing the generalizability. As the findings rely on both spatial and multilevel mixed-effect logistic regression analyses, they might help government and programme planners in developing geographic, individual, and community-focused public health interventions to improve women's comprehensive knowledge of HIV/AIDS. However, this finding should be considered in light of some limitations. Since the EDHS data are cross-sectional, determining a temporal/causal relationship is difficult, and the findings may be subject to recall- and social desirability biases.

Conclusion

About half of women in Ethiopia had inadequate HIV/AIDS comprehensive knowledge, with significant spatial variation across regions identified. Higher inadequate HIV/AIDS knowledge was identified among women with the poorest wealth quintile, being a rural resident, having

no formal education, lacking autonomy in decision-making, not being exposed to media (radio and television), not having a mobile phone, and not visiting health facilities. Thus, stakeholders in the healthcare sector need to work in the provision of HIV/AIDS education tailored to women who lack formal education, live in rural areas, and live in the poorest wealth quintile. Local and international collaborations should be strengthened to increase women's autonomy by promoting gender equality and empowering women to access HIV/AIDS health information. The media agencies also prioritise their accessibility to a broader portion of the population by providing health information tailored to women of reproductive age. The identified small geographical areas in Somali and the southern parts of Afar with relatively poor knowledge of HIV/AIDS should be targeted during resource allocation and interventions as well as further research to identify unique local factors and solutions.

Abbreviations

AOR: Adjusted odds ratio; AIC: Akaike's Information Criterion; BIC: Bayesian Information Criterion; EDHS: Ethiopian Demographic Health Survey; HIV/AIDS: Human Immunodeficiency Virus Acquired Immunodeficiency Syndrome; ICC: Intraclass Correlation Coefficient; PCV: Proportionate Change in Variance; SSA: Sub-Saharan African; WHO: World Health Organization

Acknowledgments

We would like to acknowledge the the DHS office to access the data based on a reasonable request.

Funding: Not Applicable

Data availability

The data for this study were obtained from the DHS program with a reasonable request. Thus, the one who needs the data supporting the findings of this study can get it in anonymised form from the DHS website at https://dhsprogram.com/Countries/ upon reasonable request in the same manner as the authors did.

Disclosure

We would like to assure you that, with full responsibility and communication, the document has been submitted. We indicated that there is no conflict of interest with other individuals or organizations that could influence or bias the content of the paper inappropriately

Supporting information

S1File: The most likely SaTScan clusters of areas with a high prevalence of inadequate knowledge on HIV transmission among women in Ethiopia, EDHS 2016.

References

1. *World Health Organization, HIV and AIDS, Key facts 13 July 2023 https://www.who.int/news-room/fact-sheets/detail/hiv-aids.*
2. *EPHI, HIV/AIDS National Strategic Plan for Ethiopia 2021-2025, https://www.prepwatch.org/wp-content/uploads/2022/07/Ethiopia-HIVAIDS-National-Strategic-Plan-2021-25.pdf.*
3. Dzadey, D., R.K. Biswas, and J. Bhowmik, *Investigating factors affecting HIV/AIDS knowledge among women in low and middle-income countries in Asia.* Journal of Health Psychology, 2023. **28**(11): p. 1085-1098.
4. Warren, C.E., et al., *Health systems and the SDGs: lessons from a joint HIV and sexual and reproductive health and rights response.* Health policy and planning, 2017. **32**(suppl_4): p. iv102-iv107.
5. Chan, B.T. and A.C. Tsai, *HIV knowledge trends during an era of rapid antiretroviral therapy scale-up: an analysis of 33 sub-Saharan African countries.* Journal of the International AIDS Society, 2018. **21**(7): p. e25169.
6. Sohn, A. and S. Park, *HIV/AIDS knowledge, stigmatizing attitudes, and related behaviors and factors that affect stigmatizing attitudes against HIV/AIDS among Korean adolescents.* Osong public health and research perspectives, 2012. **3**(1): p. 24-30.
7. Genberg, B.L., et al., *A comparison of HIV/AIDS-related stigma in four countries: Negative attitudes and perceived acts of discrimination towards people living with HIV/AIDS.* Social science & medicine, 2009. **68**(12): p. 2279-2287.
8. Mumtaz, G.R., et al., *Characterising HIV/AIDS knowledge and attitudes in the Middle East and North Africa: Systematic review and data synthesis.* Global public health, 2020. **15**(2): p. 275-298.
9. Yang, F., et al., *Assessment of knowledge of HIV/AIDS and association with socioeconomic disparities among young women in low-and middle-income Countries, 2003 to 2018.* JAMA Network Open, 2021. **4**(1): p. e2035000-e2035000.
10. Lan, G.L., et al., *Social capital and quality of life among people living with HIV/AIDS in Southeast China.* Asia Pacific J Public Health, 2016. **28**.
11. Snelling, D., et al., *HIV/AIDS knowledge, women's education, epidemic severity and protective sexual behaviour in low-and middle-income countries.* Journal of Biosocial Science, 2007. **39**(3): p. 421-442.
12. Chopra, N.K., H. Ni, and V. Lim, *Past present and future status of HIV-AIDS pandemic problem in world.* Microbiol Infect Dis, 2019. **3**(1): p. 1-6.
13. Egger, M., et al., *Bias in meta-analysis detected by a simple, graphical test.* Bmj, 1997. **315**.
14. Drummond, D.B.a.P.D., *Knowledge of HIV and AIDS in women in sub-Saharan Africa Amy, 2010.*
15. Burgoyne, A.D. and P.D. Drummond, *Knowledge of HIV and AIDS in women in sub-Saharan Africa.* African Journal of Reproductive Health, 2008. **12**(2): p. 14-31.
16. Teshale, A.B., et al., *Comprehensive knowledge about HIV/AIDS and associated factors among women of reproductive age in sub-Saharan Africa: a multilevel analysis using the most recent demographic and health survey of each country.* BMC Infectious Diseases, 2022. **22**(1): p. 130.
17. Ankunda, D., *Determinants of comprehensive knowledge of HIV/AIDS among women of the reproductive age (15-49) in Uganda.* 2017.
18. Gurmu, E. and D. Etana, *HIV/AIDS knowledge and stigma among women of reproductive age in Ethiopia.* African Journal of AIDS Research, 2015. **14**(3): p. 191-199.
19. Assefa, Y., et al., *Towards achieving the fast-track targets and ending the epidemic of HIV/AIDS in Ethiopia: successes and challenges.* International Journal of Infectious Diseases, 2019. **78**: p. 57-64.

20. Agegnehu, C.D., et al., *Determinants of comprehensive knowledge of HIV/AIDS among reproductive age (15–49 years) women in Ethiopia: further analysis of 2016 Ethiopian demographic and health survey.* AIDS Research and Therapy, 2020. **17**: p. 1-9.
21. Hays, R.D., et al., *Health-related quality of life in patients with human immunodeficiency virus infection in the United States: results from the HIV Cost and Services Utilization Study.* Am J Med, 2000. **108**.
22. *CSA, I., Central statistical agency (CSA)[Ethiopia] and ICF. Ethiopia demographic and health survey, Addis Ababa, Ethiopia and Calverton, Maryland, USA, 2016. 1. https://dhsprogram.com/pubs/pdf/FR328/FR328.pdf.*
23. Agegnehu, C.D. and G.A. Tesema, *Effect of mass media on comprehensive knowledge of HIV/AIDS and its spatial distribution among reproductive-age women in Ethiopia: a spatial and multilevel analysis.* BMC public health, 2020. **20**(1): p. 1-12.
24. Liyeh, T.M., et al., *Knowledge of prevention of mother to child transmission of HIV among women of reproductive age group and associated factors at Mecha district, Northwest Ethiopia.* BMC Research Notes, 2020. **13**(1): p. 1-6.
25. Kassie, S.Y., A.A. Chereka, and Y. Damtie, *Systematic review and meta-analysis of knowledge on PMTCT of HIV/AIDS and Association factors among reproductive age women in Ethiopia, 2022.* BMC Infectious Diseases, 2023. **23**(1): p. 491.
26. Raza, O., et al., *Geographically weighted regression analysis: A statistical method to account for spatial heterogeneity.* Archives of Iranian Medicine, 2019. **22**(3): p. 155-160.
27. Detels, R., et al., *Effectiveness of potent antiretroviral therapy on time to AIDS and death in men with known HIV infection duration. Multicenter AIDS Cohort Study Investigators.* Jama, 1998. **280**.
28. Gange, S.J., et al., *Effectiveness of highly active antiretroviral therapy among HIV-1 infected women.* J Epidemiol Community Health, 2002. **56**.
29. Iqbal, S., et al., *Determinants of overall knowledge of and attitudes towards HIV/AIDS transmission among ever-married women in Pakistan: evidence from the Demographic and Health Survey 2012–13.* BMC public health, 2019. **19**(1): p. 1-14.
30. Ciampa, P.J., et al., *Comprehensive knowledge of HIV among women in rural Mozambique: development and validation of the HIV knowledge 27 scale.* PloS one, 2012. **7**(10): p. e48676.
31. Ochako, R., et al., *Trends and determinants of Comprehensive HIV and AIDS knowledge among urban young women in Kenya.* AIDS research and therapy, 2011. **8**(1): p. 1-8.
32. Son, N.V., et al., *Trends and factors associated with comprehensive knowledge about HIV among women in Vietnam.* Tropical Medicine and Infectious Disease, 2020. **5**(2): p. 91.
33. Asabu, M.D. and D.K. Altaseb, *The trends of women's autonomy in health care decision making and associated factors in Ethiopia: evidence from 2005, 2011 and 2016 DHS data.* BMC Women's Health, 2021. **21**: p. 1-9.
34. Habte, A., A. Tamene, and B. Bogale, *Women empowerment domains and unmet need for contraception among married and cohabiting fecund women in Sub-Saharan Africa: A multilevel analysis based on gender role framework.* Plos one, 2023. **18**(9): p. e0291110.
35. Becker, S. and A. Kalamar, *Sampling weights for analyses of couple data: Example of the Demographic and Health Surveys.* Demography, 2018. **55**(4): p. 1447-1473.
36. Kissling, W.D. and G. Carl, *Spatial autocorrelation and the selection of simultaneous autoregressive models.* Global Ecology and Biogeography, 2008. **17**(1): p. 59-71.
37. Ord, J.K. and A. Getis, *Local spatial autocorrelation statistics: distributional issues and an application.* Geographical analysis, 1995. **27**(4): p. 286-306.
38. Ahdieh, L., et al., *Selection by indication of potent antiretroviral therapy use in a large cohort of women infected with human immunodeficiency virus.* Am J Epidemiol, 2000. **152**.
39. Gribov, A. and K. Krivoruchko, *Empirical Bayesian kriging implementation and usage.* Science of the Total Environment, 2020. **722**: p. 137290.

40. Warden, C.R., *Comparison of Poisson and Bernoulli spatial cluster analyses of pediatric injuries in a fire district.* International Journal of Health Geographics, 2008. **7**: p. 1-17.
41. Kulldorff, M., *SaTScanTM user guide https://www.satscan.org/cgi-bin/satscan/register.pl/Current%20Version:%20SaTScan%20v9.2%20released%20October%2022%202013.?todo=process_userguide_download*. 2006, Boston.
42. Manepalli, U., G.H. Bham, and S. Kandada. *Evaluation of hotspots identification using kernel density estimation (K) and Getis-Ord (Gi*) on I-630*. in *3rd International Conference on Road Safety and Simulation*. 2011. National Academy of Sciences Indianapolis Indiana, United States.
43. Songchitruksa, P. and X. Zeng, *Getis–Ord spatial statistics to identify hot spots by using incident management data.* Transportation research record, 2010. **2165**(1): p. 42-51.
44. Wang, H. and J. Zhu, *Variable selection in spatial regression via penalized least squares.* Canadian Journal of Statistics, 2009. **37**(4): p. 607-624.
45. Beale, C.M., et al., *Regression analysis of spatial data.* Ecology letters, 2010. **13**(2): p. 246-264.
46. Chi, G. and J. Zhu, *Spatial regression models for demographic analysis.* Population Research and Policy Review, 2008. **27**: p. 17-42.
47. Estifanos, T.M., et al., *Predictors of HIV/AIDS comprehensive knowledge and acceptance attitude towards people living with HIV/AIDS among unmarried young females in Uganda: a cross-sectional study.* BMC Women's Health, 2021. **21**: p. 1-13.
48. Efendi, F., et al., *HIV-related knowledge level among Indonesian women between 15 years and 49 years of age.* African Health Sciences, 2020. **20**(1): p. 83-90.
49. Zhang, T., et al., *Awareness of HIV/AIDS and its routes of transmission as well as access to health knowledge among rural residents in Western China: a cross-sectional study.* BMC public health, 2019. **19**(1): p. 1-11.
50. Zainiddinov, H. and N. Habibov, *Trends and predictors of knowledge about HIV/AIDS and its prevention and transmission methods among women in Tajikistan.* The European Journal of Public Health, 2016. **26**(6): p. 1075-1079.
51. Teshome, R., et al., *Comparison and association of comprehensive HIV/AIDS knowledge and attitude towards people living with HIV/AIDS among women aged 15-49 in three east African countries: Burundi, Ethiopia and Kenya.* J AIDS Clin Res, 2016. **7**(4): p. 1-8.
52. Malik, M., et al., *Knowledge of HIV/AIDS and its determinants in India: Findings from the National Family Health Survey-5 (2019–2021).* Population Medicine, 2023. **5**(May): p. 1-12.
53. Siziya, S., A.S. Muula, and E. Rudatsikira, *HIV and AIDS-related knowledge among women in Iraq.* BMC research notes, 2008. **1**(1): p. 1-6.
54. Mandiwa, C., B. Namondwe, and M. Munthali, *Prevalence and correlates of comprehensive HIV/AIDS knowledge among adolescent girls and young women aged 15–24 years in Malawi: evidence from the 2015–16 Malawi demographic and health survey.* BMC Public Health, 2021. **21**(1): p. 1-9.
55. Gari, S., et al., *Access to HIV/AIDS care: a systematic review of socio-cultural determinants in low and high income countries.* BMC health services research, 2013. **13**(1): p. 1-13.
56. Bekalu, M.A., S. Eggermont, and K.V. Viswanath, *HIV/AIDS communication inequalities and associated cognitive and affective outcomes: A call for a socioecological approach to AIDS communication in Sub-Saharan Africa.* Health communication, 2017. **32**(6): p. 685-694.
57. Kefale, B., et al., *Predictors of comprehensive knowledge of HIV/AIDS among people aged 15–49 years in Ethiopia: a multilevel analysis.* HIV/AIDS-Research and Palliative Care, 2020: p. 449-456.
58. Sundaram, M.S., M. Sekar, and A. Subburaj, *Women empowerment: role of education.* International Journal in Management & Social Science, 2014. **2**(12): p. 76-85.
59. Chirwa, G.C., *"Who knows more, and why?" Explaining socioeconomic-related inequality in knowledge about HIV in Malawi.* Scientific African, 2020. **7**: p. e00213.

Click here to access/download;Figure;Fig 1.tif

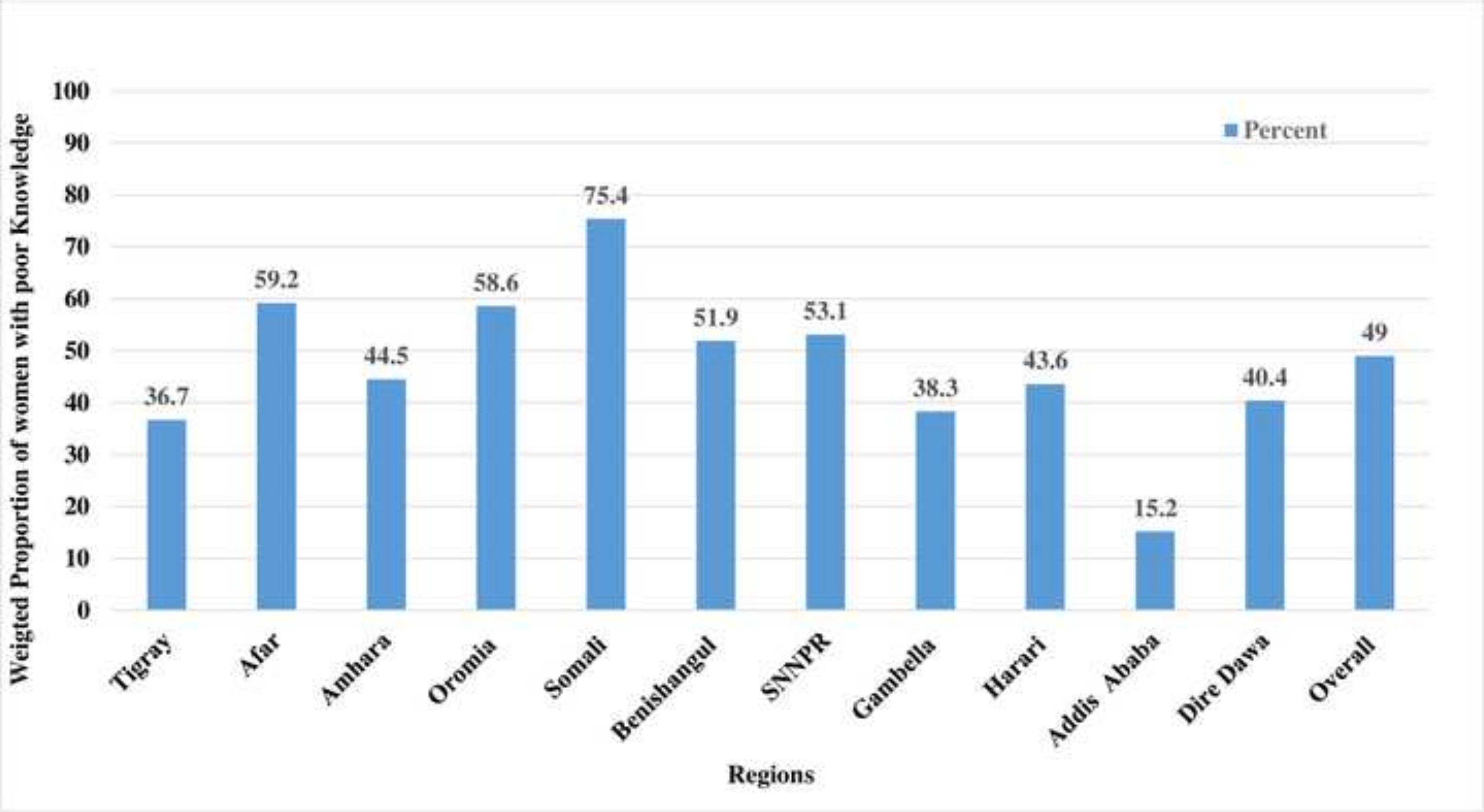

Click here to access/download;Figure;Fig 2.tif

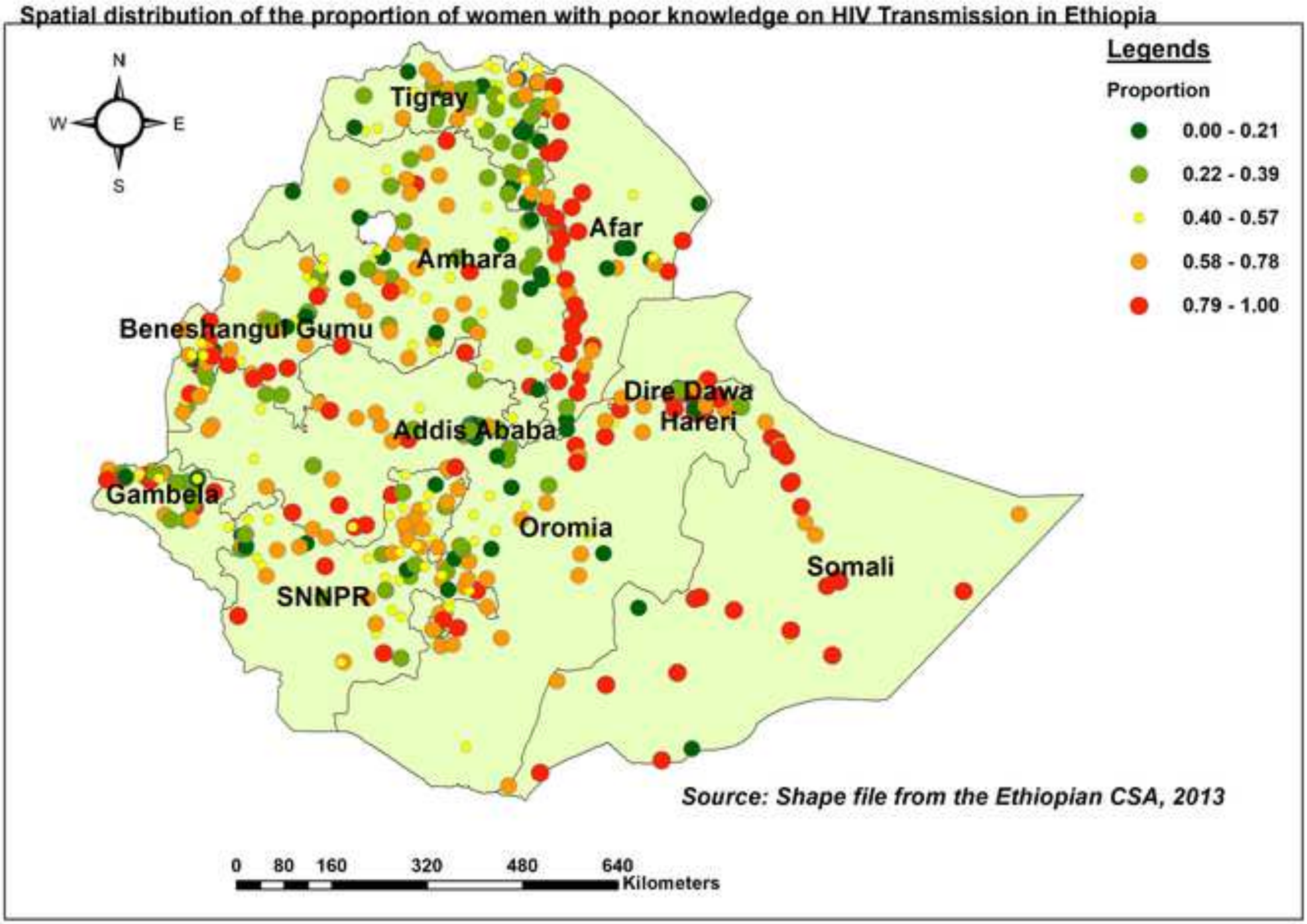

Click here to access/download;Figure;Fig 3.tif

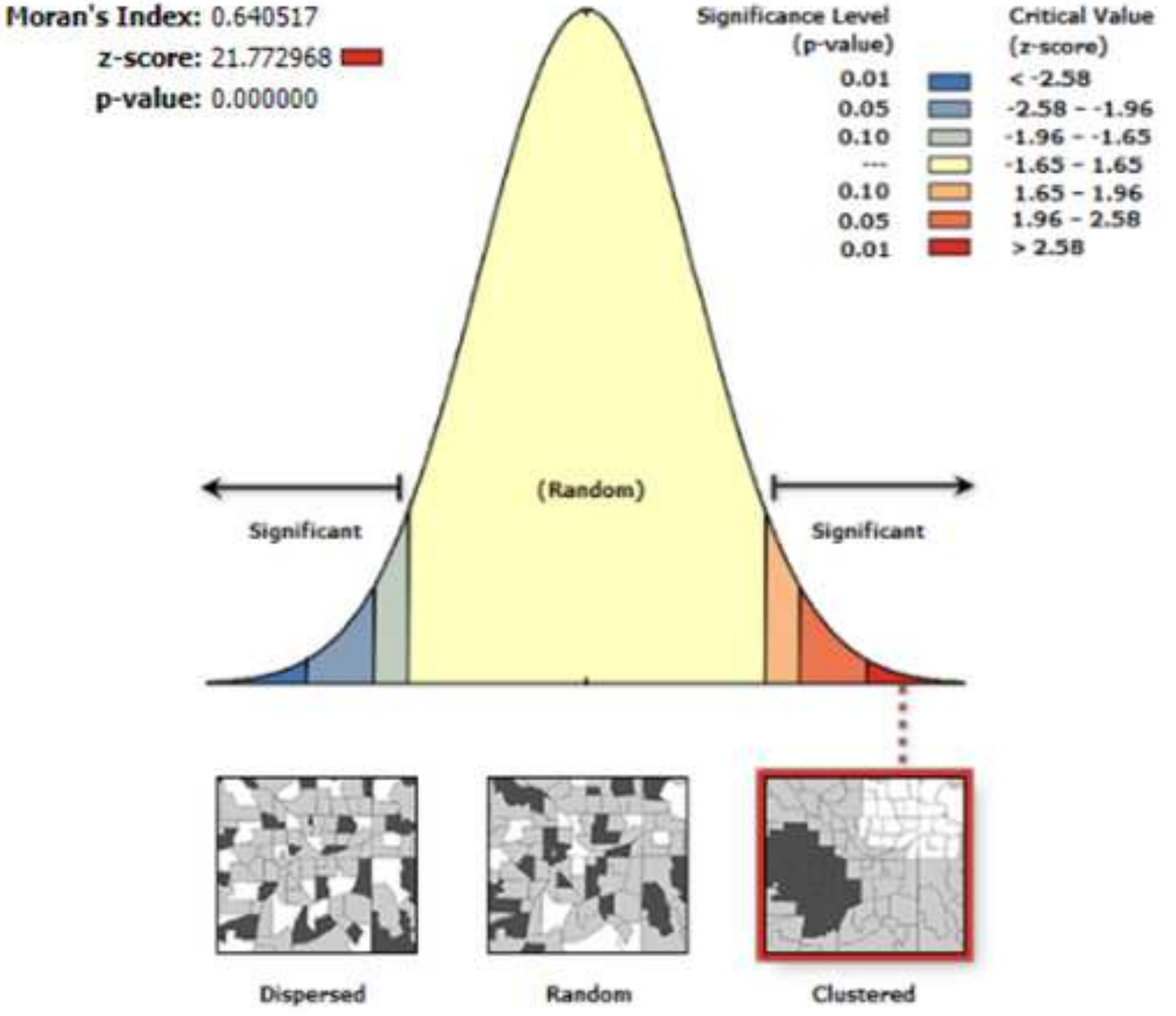

Given the z-score of 21.7729675174, there is a less than 1% likelihood that this clustered pattern could be the result of random chance.

Click here to access/download;Figure;Fig 4.tif

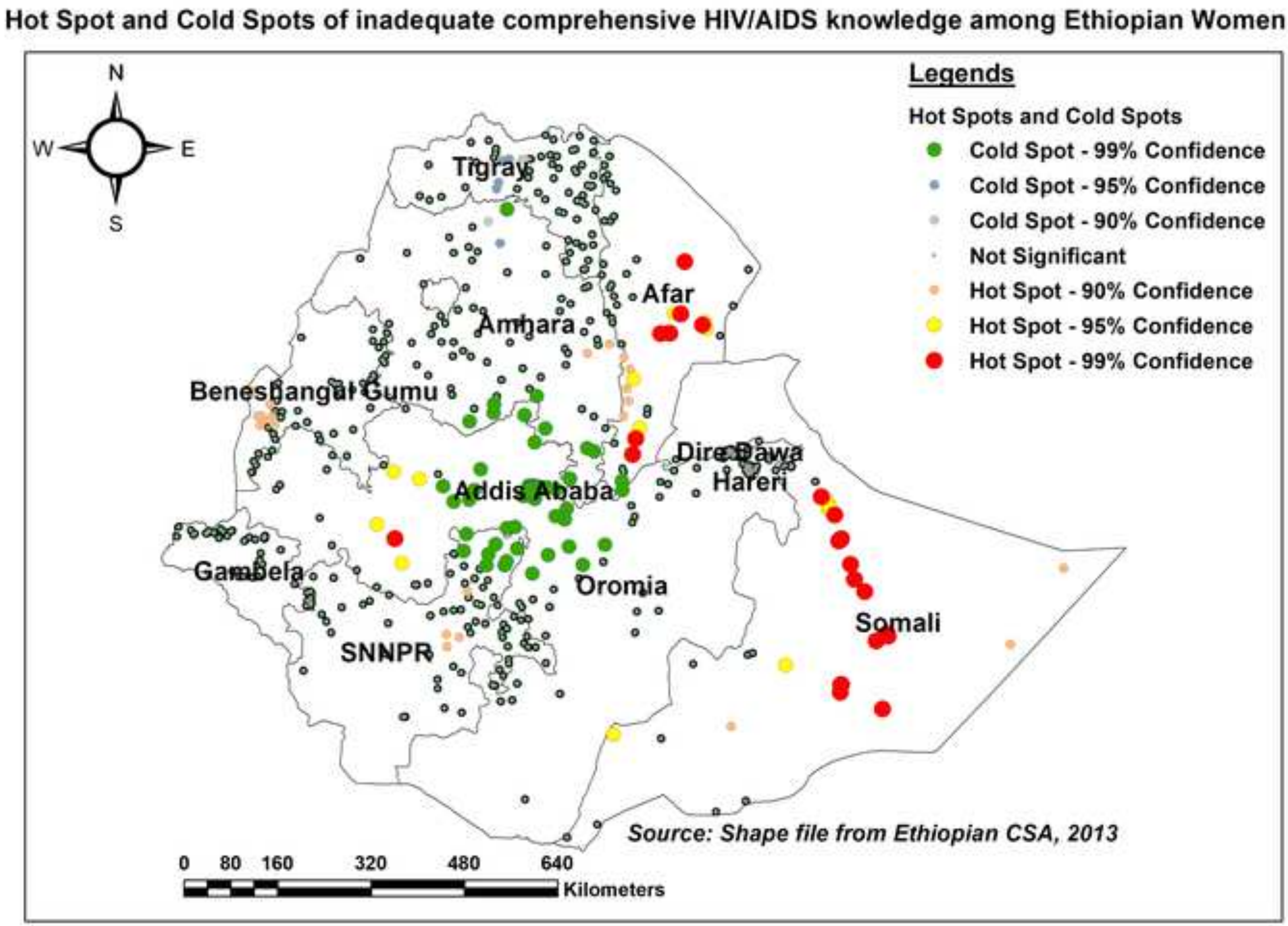

Click here to access/download;Figure;Fig 5.tif

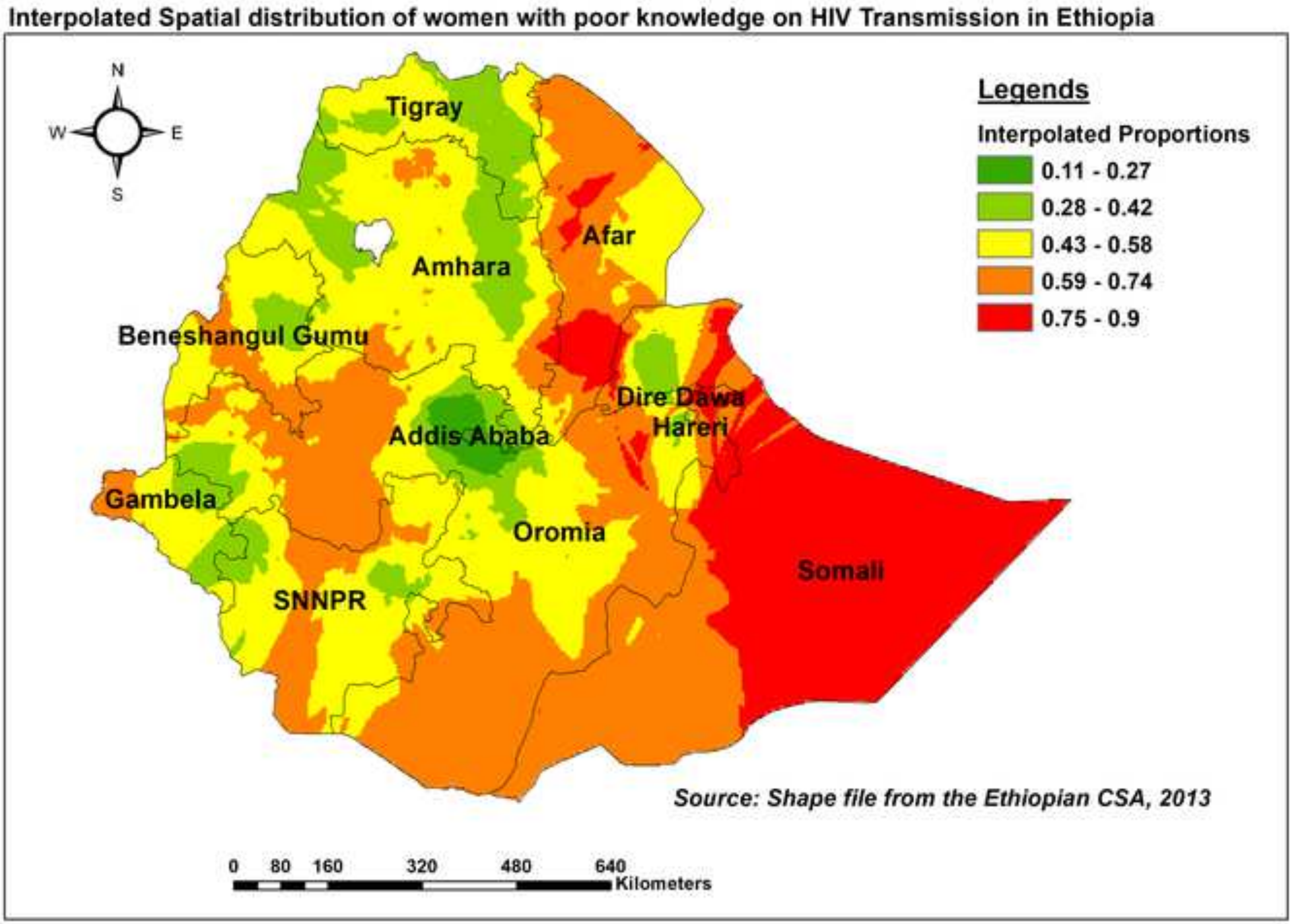

Click here to access/download;Figure;Fig 6.tif

GWR analysis for spatial predictors of having inadequate knowledge on HIV transmission among women in Ethiopia

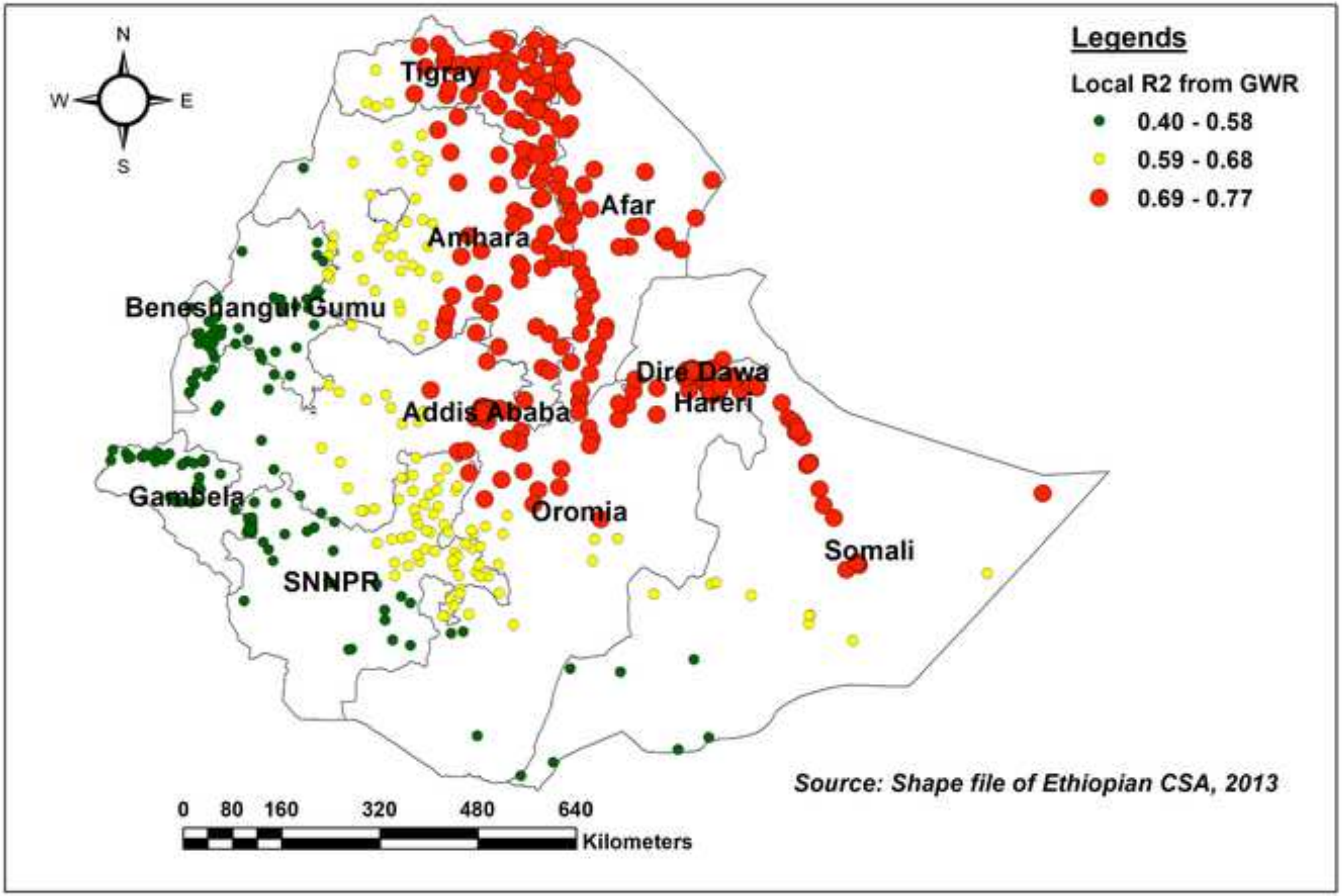

Proportion of women without formal education

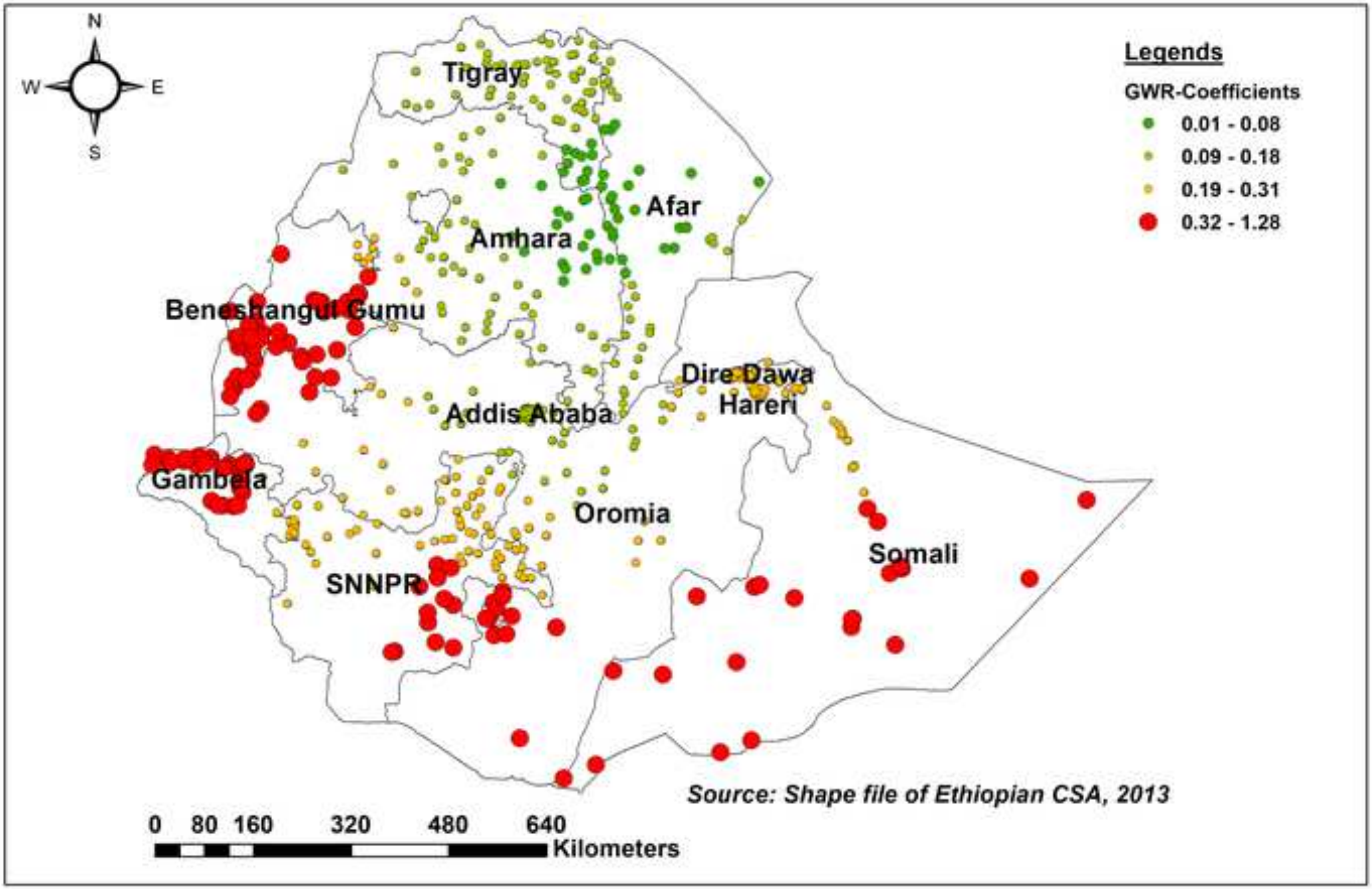

Click here to access/download;Figure;Fig 8.tif

GWR analysis for spatial association between living in the poorest wealth quintile and inadequate knowledge

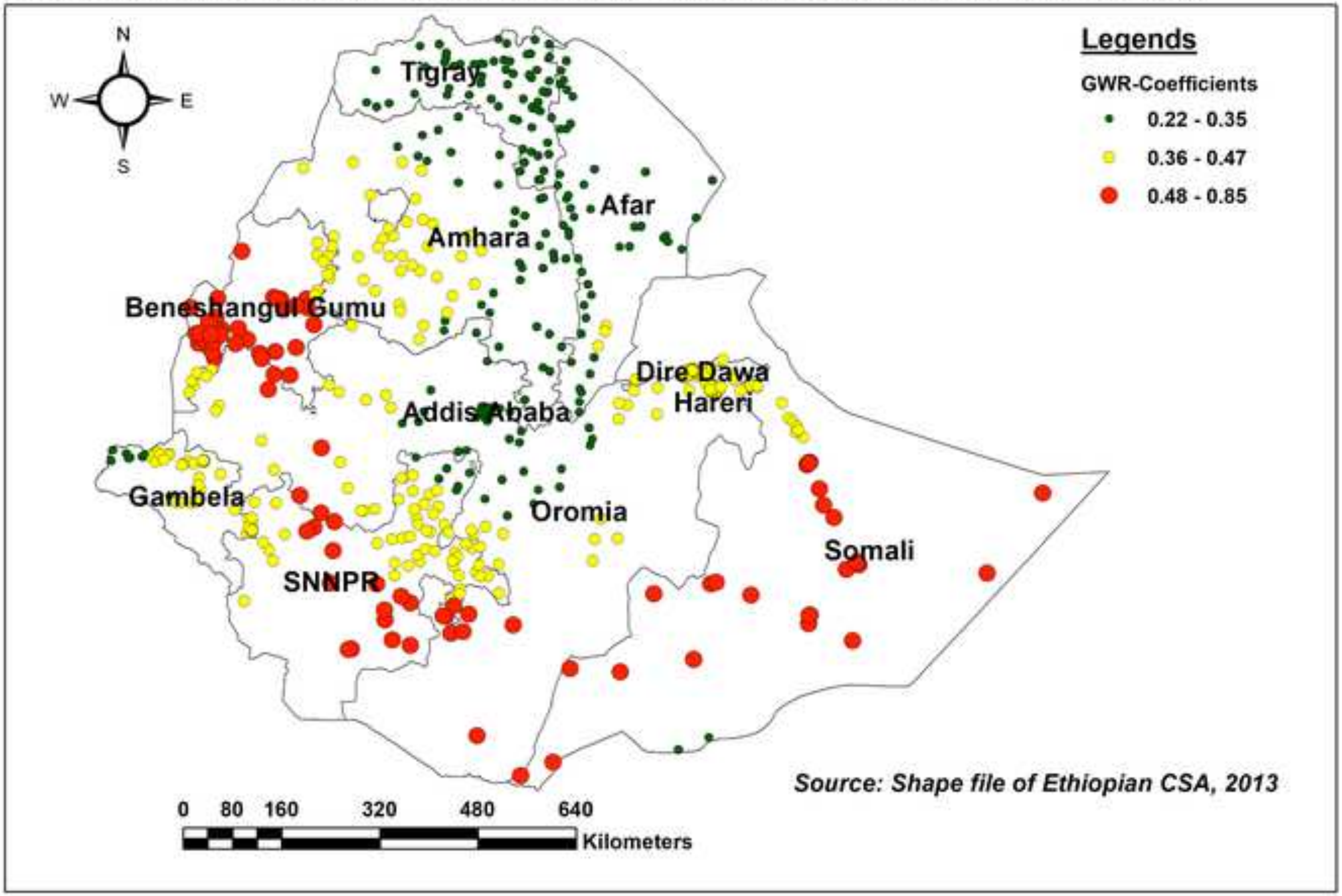

Click here to access/download;Figure;Fig 9.tif

GWR analysis for spatial association between being non-autonomous and inadequate knowledge of HIV transmission

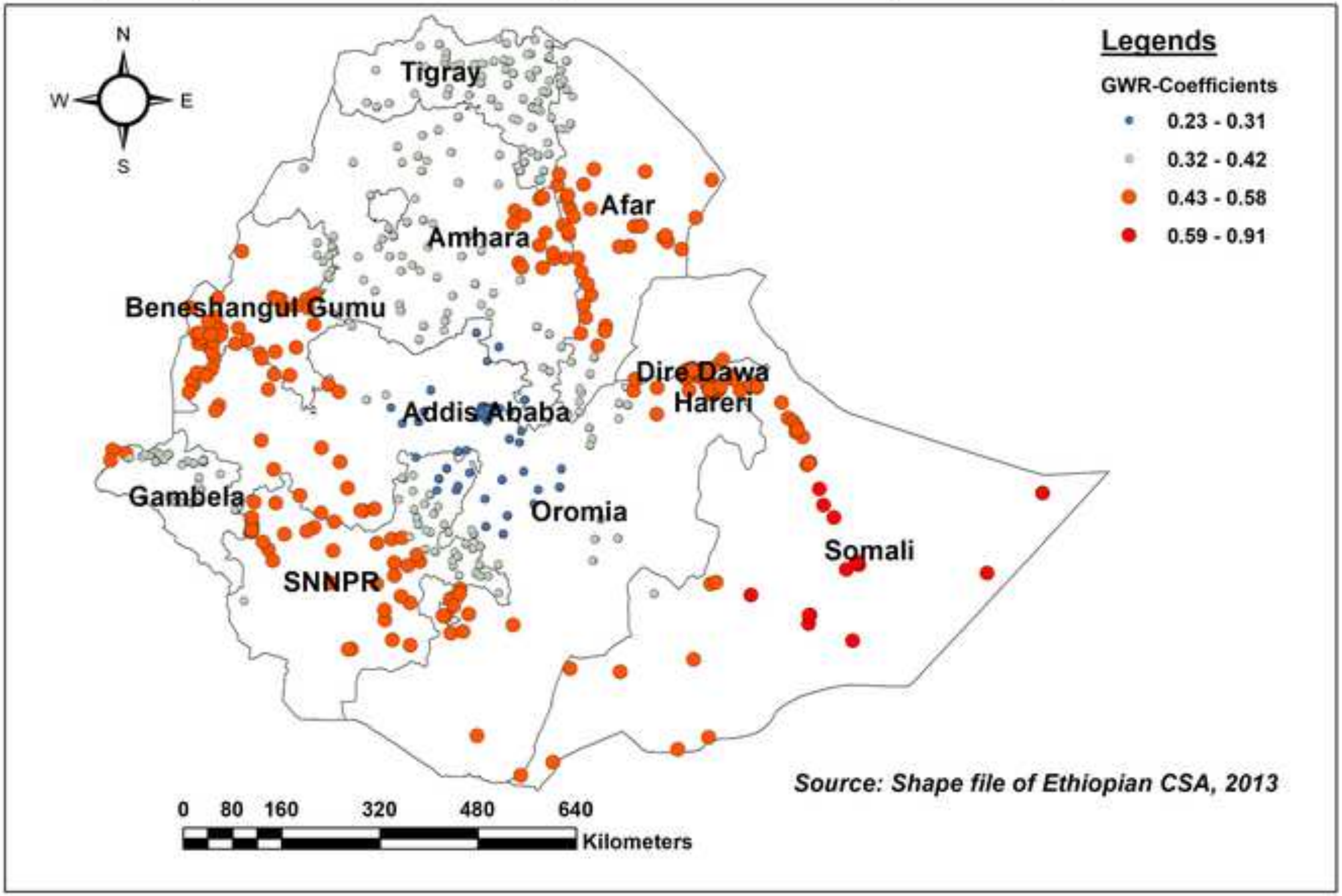

Click here to access/download;Figure;Fig 10.tif

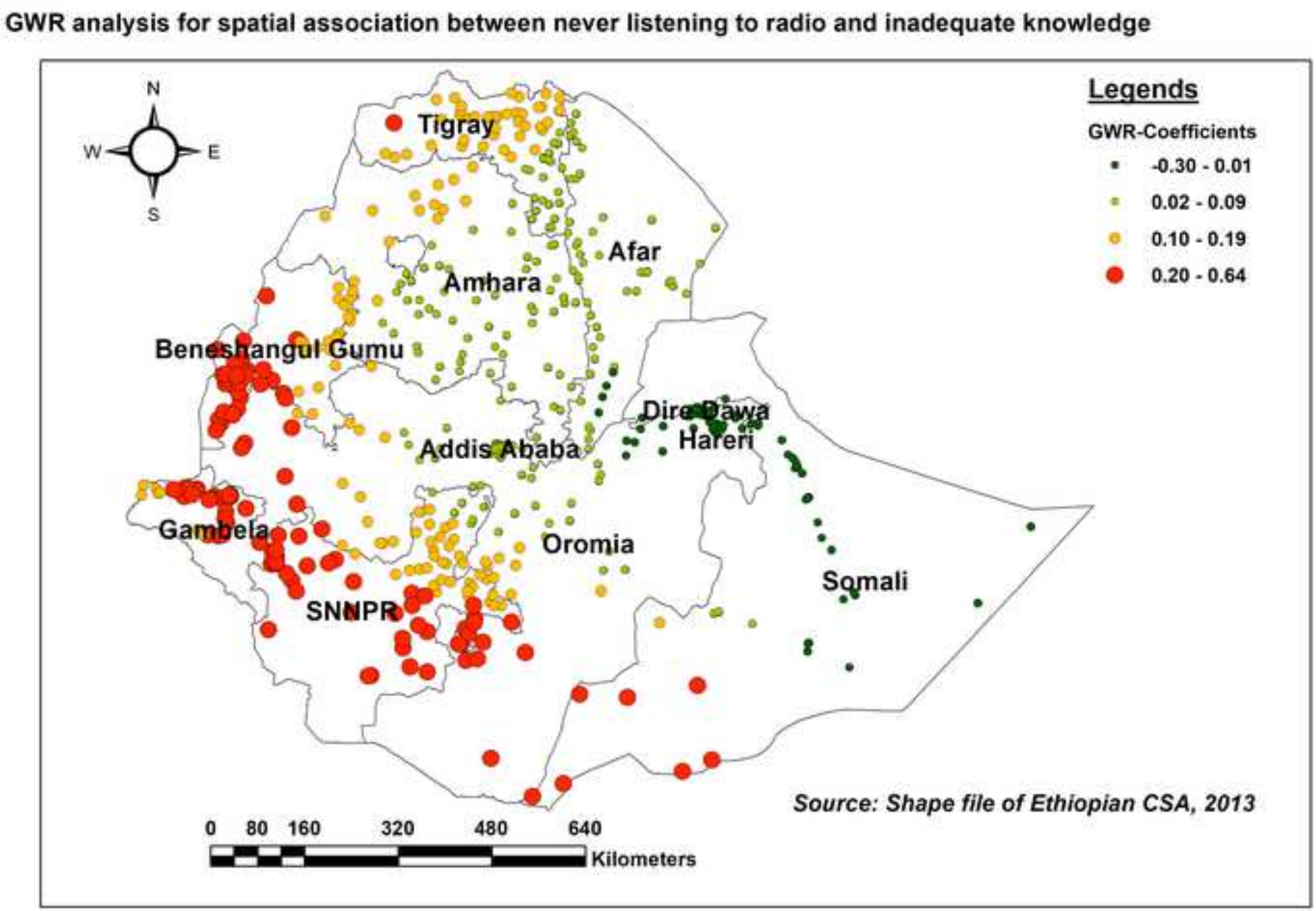

Click here to access/download;Figure;Fig 11.tif

GWR analysis for spatial association between never watching TV and inadequate knowledge

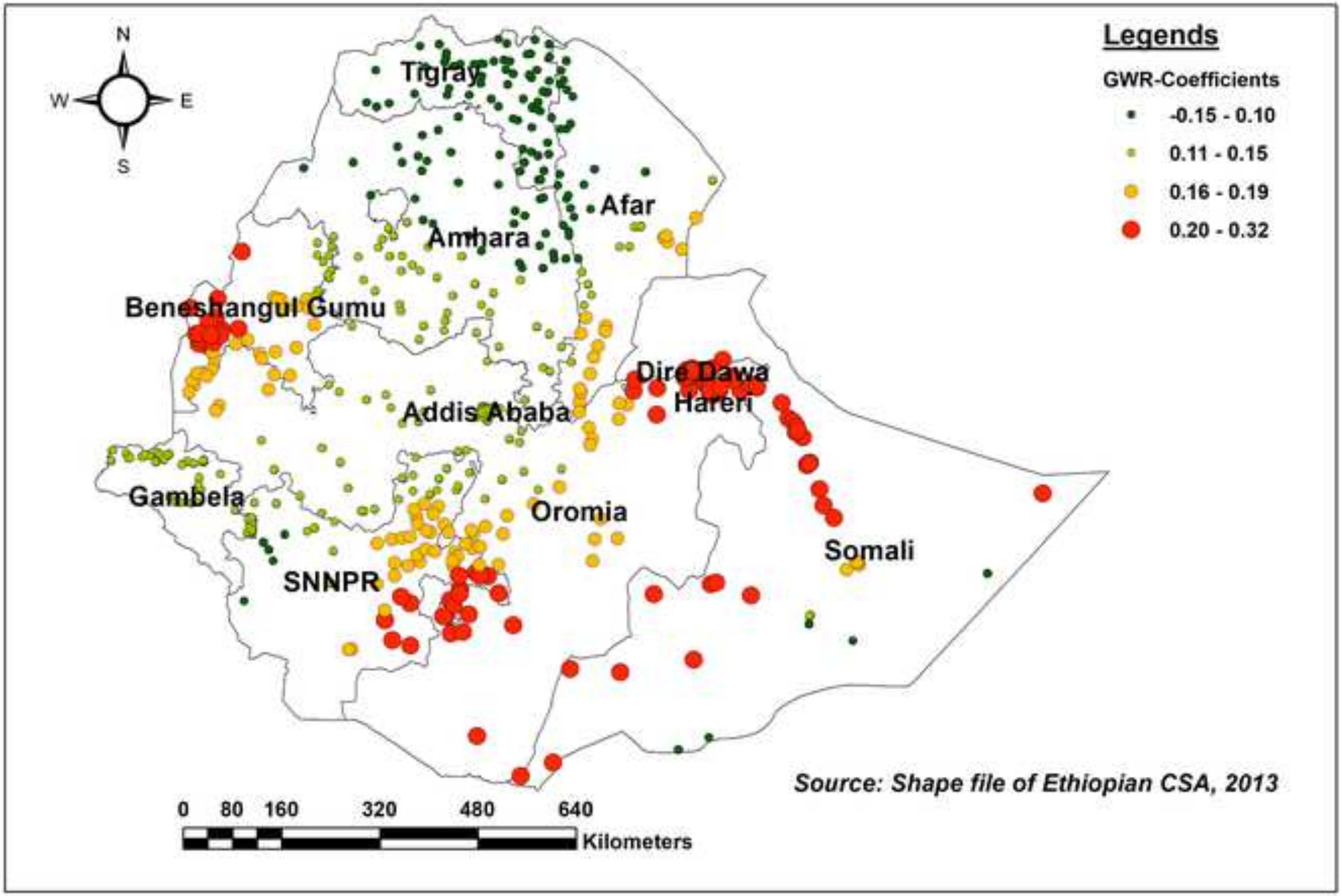

Click here to access/download;Figure;Fig 12.tif

GWR analysis for spatial association between not visiting health facility and inadequate knowledge

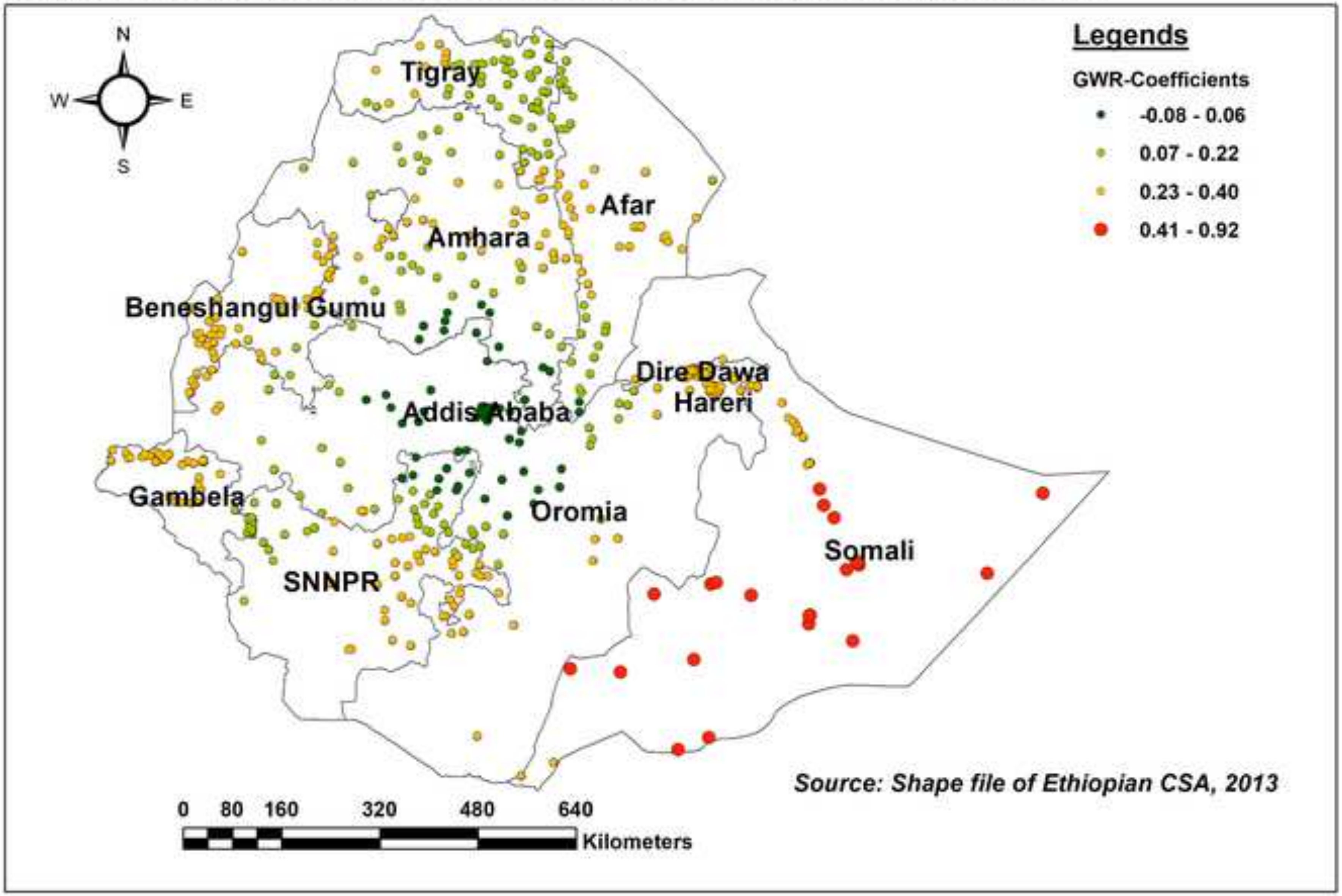

Click here to access/download
Supporting Information
S1File.docx
