# Supplementary material for: Spatial variation and predictors of composite index of HIV/AIDS knowledge, attitude and behaviours among Ethiopian women: A spatial and multilevel analyses of the 2016 Demographic Health Survey
Source: PLoS One. 2024 Jun 4;19(6):e0304982. doi: 10.1371/journal.pone.0304982 (PMC11149886; doi:10.1371/journal.pone.0304982)
Supplement: Attachment — Submitted filename: Peer review for HIV knowledge.docx [file pone.0304982.s004.docx]

**Abstract**

The abstract section you provided appears to be generally well-written and provides a clear overview of the study's objectives, methods, and findings. However, there are a few areas where the abstract could be improved:

Background: It would be helpful to include more detailed information in the background section regarding the research that have been done on Ethiopian women's awareness of HIV/AIDS. It would be useful to specify the number of studies, their scope, and any important conclusions or limits rather than just saying that "some studies" were undertaken. If your goal is to investigate the gaps in your knowledge, then please be content with that.

Objective: Where are the predictors found by the regression analysis in this case that the author stated, "Consequently, this study attempted to assess the prevalence, geographical variation (Hotspots), spatial predictors, and multilevel correlations of inadequate HIV/AIDS awareness among Ethiopian women"? Because of this, the spatial regression method was used to identify your predictors instead of the multilevel regression analysis. What does little understanding of HIV/AIDS mean in terms of multilayer correlates? The writers must choose the appropriate regression model and have a well-defined target.

Methods: Although the study's use of data from the 2016 Ethiopian Demographic and Health Survey is noted, it would be beneficial to include a brief overview of the survey's methodology and sampling plan. This would provide readers a clearer picture of the sample's representativeness and the source of the data.

Results: The prevalence of inadequate knowledge about HIV/AIDS among Ethiopian women is presented in the abstract; nonetheless, it would be helpful to give a brief overview of the main multilevel and spatial correlates of inadequate knowledge. This would help readers understand the causes of insufficient knowledge and the extent of their impacts.

Which source did you use to get the reference group variables?

Conclusion: Expanding the conclusion section to include more detailed suggestions based on the data would be beneficial. For instance, it might outline the precise treatments or tactics that could be used to close the observed knowledge gaps, rather than just recommending the need for customized health education and awareness campaigns.

**Introduction**

The introduction section you provided contains valuable information about the global and Ethiopian context of HIV/AIDS and the importance of comprehensive knowledge for prevention efforts. However, there are a few areas where the introduction could be improved:

**Structure:** There is room for improvement in the introduction's structure. Think about breaking up the section into paragraphs that each focus on a different facet of the subject. The content would flow more easily for readers as a result.

**Citations:** It would be beneficial to give precise citations for each piece of material, even though you have included some references to back up the claims stated in the introduction. This will guarantee the accuracy of the material provided and allow readers the chance to go further into the research that are cited for further details.

**Clarity and Conciseness:** The introduction has a few long and complicated sentences that may be challenging to grasp. To make the text easier to understand, try to make the language simpler and divide longer, more complicated statements into shorter, more direct ones.

**Research Gap:** Although the introduction notes that earlier research on Ethiopian women's comprehensive knowledge of HIV/AIDS was small in scale and scope, it would be helpful to highlight the particular research gap that this study attempts to fill. Indicate exactly how this study is different from the others and what special contributions it will make. In comparison to other earlier studies, the research gap was not often addressed or stated very clearly.

**Significance:** The study's possible ramifications for community empowerment, policy decisions, and focused interventions are touched upon in passing in the introduction. Explain the wider significance of filling the identified research gap as well as any possible practical ramifications in order to strengthen this section.

The introductory section can be improved to give a more condensed and targeted summary of the background, purpose, and importance of the study by taking these ideas into consideration.

Regarding SDG 3, the references 3 and 4 were incorrect. Kindly make use of the primary source for your information.

Some of the references are outdated (e.g. reference 13 was published since 1997)

Although the author claimed that references 20 and 23–25 were narrow in scope and did not examine the elements at the person and community levels, these references are not as narrow as the author claimed.

There are instances of misspellings, acronyms, and capitalizations throughout the entire document. Therefore, kindly make the necessary revisions.

**Methods**

First create an independent section about the study setting, and period, before you go to the data and population section.

**Data and population**

Women who have missing data about their residential area locations via the geographical positioning system (GPS) were excluded from the spatial analysis. How did you do it? Does this meet the proper exclusion criteria for your study over the other criteria? Create an independent section titled “Source and Study Population," and then you might narrate the criteria for your study.

**Sampling procedures**

Please kindly include more depth information about it for readers. We did not expect to ready your references always. Put them according to the previous studies or the DHS information (for illustration urban versus rural household allocations, and total EAs in Ethiopia were not addressed). Furthermore, the authors did not inform us about data collection tools, however, they put it in the title as a subsection in the methods

References 21, 27, and 28 are not your appropriate evidence. Moreover, the publication data (1998) and the information inside it are quite different with regard to your interest. Kindly revise them.

**Measurement of variables of the study**

The author should see it again in this section. First, the outcome ascertainment should be based on the guide to DHS, not on the interest of the author. Second, utilizing “mean” on compressive knowledge is not recommended by the guidelines; however, the author did it. Why? Based on what criteria or reference they used, Third, adequate and inadequate categorization should be read again by the author. Fourth, there are also attitude- or practice-related questions in their tool; however, they treated them for knowledge, and this should be taken into consideration. Finally, the author provided a reference for their outcome classification (reference 29); however, this reference was not the right reference for the outcome of interest. Outcome ascertainment is the heart of every research project and should be corrected properly before going to the next step.

**Explanatory variables**

The author missed some important variables such as working or employment status, ANC, place of delivery, health visits, modern contraceptive utilization (the given contraceptive utilization should be clearly defined rather than using any methods), sex of the given household head, breastfeeding status, and other individual-level factors. Fortunately, all these variables are available in the EDHS 2016 dataset. Furthermore, the author failed to include important community-level factors, such as community women's wealth, community women's education level, community-level ANC coverage, community-level women's mass media exposure, and so on. Kindly include all these and other important variables in your outcome.

Variable selection and categorization should be done in an acceptable manner. For instance, media exposure was better classified into either of the three types (TV, radio, or newspapers). Having a mobile phone or not will not have a great implication in the context of Ethiopia. (Assume your data collection period.)

**Data management and statistical analysis**

Why used weighting factor v005/1000000, why not other numbers like 1000, or 10000000

If you used GWR, why did you use a multilevel analysis again? or you did not do GWR. The GWR is the regression component for the spatial analysis, and if you applied the GWR, then your probability of coming back again to the multilevel analysis is not feasible. Spatial and multilevel analyses are quite different approaches, and the best regression for you is GWR rather than GLMM.

Use associated factors, or determinants instead of predictors

May the author show us the dataset of outcome variable ascertainments, and the performance of the OLS, and GWR regressions or the do file?

Use the phrase multilevel or mixed effect analysis separately, they are the same thing. Don’t use them as multilevel mixed effect analysis

The outcome variable categorization should be the same for the spatial and regression analyses, either inadequate or adequate knowledge about HIV/AIDS

Use either AIC, or BIC. These two are have different assumptions and working correlation regarding the complexity and parameters of the given model

Although the data has missing values, the author failed to inform how they managed the missing values.

**Results**

Somalia Vs Somali, should be corrected

The results sections seem discussion due its lot interpretation

Put number before the percentage. For instance, (876 (34.3%)

There is inconsistency of variables utilization throughout the manuscript. Variables from the regression table should be incorporated in the descriptive sections first

Put the PCV, and MOR

Please interpret and talk about the OLS model Diagnosis in your article

Explain the random variation in better please

Use either AOR or aOR, including the abstract section

**Discussion**

Overall, the discussion section provides a comprehensive analysis of the study's findings and their implications. However, there are a few areas where the discussion could be improved:

**Interpretation of Findings:** The discussion primarily presents the findings without delving into their underlying causes or providing in-depth interpretations. For example, when discussing the determinants of inadequate comprehensive knowledge, it would be beneficial to explore the mechanisms through which factors such as education, wealth quintile, autonomy, and media exposure influence knowledge levels. Providing a more nuanced understanding of these relationships would enhance the discussion.

**Comparison with Other Studies:** The discussion includes comparisons with studies conducted in other countries, highlighting differences in knowledge levels. However, it would be helpful to provide more context by discussing potential reasons for the variations observed. What are the specific factors that contribute to higher or lower knowledge levels in different countries? This would allow for a more meaningful comparison and a deeper understanding of the factors influencing knowledge levels.

**Implications and Recommendations:** While the discussion briefly mentions the need for tailored interventions and policy initiatives, it could benefit from more concrete recommendations based on the study's findings. For instance, how can public health campaigns, education programs, and policy initiatives be designed to address the identified determinants of inadequate knowledge? What specific strategies can be employed to improve access to education, healthcare, and media exposure? Providing practical recommendations would enhance the applicability of the study's findings.

**Limitations:** The discussion mentions the limitations of the study, such as the cross-sectional nature of the data and the potential for recall and social desirability biases. However, it would be beneficial to discuss these limitations in more detail and consider their impact on the study's findings. Additionally, it would be helpful to suggest avenues for future research that could address these limitations and provide a more robust understanding of HIV/AIDS knowledge among Ethiopian women.

By addressing these suggestions, the discussion section can be improved to provide a more thorough analysis of the study's findings, their implications, and recommendations for future interventions and research.

**Conclusion**

Try to include the implication of your study briefly.
