# Supplementary material for: Spatial variation and predictors of composite index of HIV/AIDS knowledge, attitude and behaviours among Ethiopian women: A spatial and multilevel analyses of the 2016 Demographic Health Survey
Source: PLoS One. 2024 Jun 4;19(6):e0304982. doi: 10.1371/journal.pone.0304982 (PMC11149886; doi:10.1371/journal.pone.0304982)
Supplement: Attachment — Submitted filename: Reviewing of paper (2 Ethiopia).docx [file pone.0304982.s006.docx]

I would like to say thank you for considering me to review your paper which is current issue entitled as “Spatial variation and predictors of inadequate HIV/AIDS knowledge among Ethiopian Women: A spatial and multilevel analyses of the 2016 Demographic Health Survey”.

Generally, it is interesting title, information is current issue in the world and the information is so far limited, however the data source is too outdated. Saying this the following massage and question should be addressed.

1. You should revise the manuscript based on Plose one manuscript preparation guideline
2. In your manuscript, you have used abbreviation in title and abstract part. Do you believe this is appropriate and advisable in scientific research journal?
3. Should write the study design clearly you used from your data source?
4. What is the importance of doing geographically weighted regression and logistic regression simultaneously? I think geographically weighted regression is strong enough to generate your evidence?

**Good lack**
