# Supplementary material for: Spatial variation and predictors of composite index of HIV/AIDS knowledge, attitude and behaviours among Ethiopian women: A spatial and multilevel analyses of the 2016 Demographic Health Survey
Source: PLoS One. 2024 Jun 4;19(6):e0304982. doi: 10.1371/journal.pone.0304982 (PMC11149886; doi:10.1371/journal.pone.0304982)
Supplement: Attachment — Submitted filename: Response to reviewers.docx [file pone.0304982.s007.docx]

**A point-by-point response to editor and reviewers**

**Authors’ Response to Academic Editor**

**Dear: Clement Ameh Yaro, Ph.D, Academic Editor, Plos One**

We thank you for a thorough reading and constructive comments and suggestions on our manuscript and for the opportunity to revise and resubmit. We are pleased to submit the revised version of the manuscript titled “**Spatial variation and predictors of inadequate HIV/AIDS knowledge among Ethiopian Women: A spatial and multilevel analyses of the 2016 Demographic Health Survey**” for your consideration in the special collection of **Plos One**. The comments of the editors and the reviewers were highly insightful and enabled us to greatly improve the quality of our manuscript. In this revised manuscript we made substantial changes to address your concerns in a point-by-point response. We appreciate your time and look forward to your response and we are very keen to incorporate further comments, if any, for the betterment of the final manuscript.

On the following pages, you will find our responses to the comments and suggestions raised by the esteemed editor and reviewer.

Sincerely,

Aklilu Habte(MPH)(corresponding author)

[**aklilihabte57@gmail.com**](mailto:aklilihabte57@gmail.com)

**Response to Journal requirements**

**1.** Please ensure that your manuscript meets PLOS ONE's style requirements, including those for file naming.

**Response:** we already prepared the manuscript as per the journal requirement and again we rechecked the compliance towards it during the submission of our revised manuscript.

**2.** For studies involving third-party data, we encourage authors to share any data specific to their analyses that they can legally distribute. PLOS recognizes, however, that authors may be using third-party data they do not have the rights to share. When third-party data cannot be publicly shared, authors must provide all information necessary for interested researchers to apply to gain access to the data. (<https://journals.plos.org/plosone/s/data-availability#loc-acceptable-data-access-restrictions>). For any third-party data that the authors cannot legally distribute, they should include the following information in their Data Availability Statement upon submission:

a) A description of the data set and the third-party source

b) If applicable, verification of permission to use the data set

c) Confirmation of whether the authors received any special privileges in accessing the data that other researchers would not have

d) All necessary contact information others would need to apply to gain access to the data

**Response:** Thank you for your suggestion. However, we already have incorporated in the initial manuscript and now we have highlighted it in the ‘Data availability’ section of the "Revised Manuscript with Track Changes", Lines 580 to 583, Page 24 for your convenience.

**3.** We note that Figures 2, 4, 5, 6, 7, 8, 9, 10, 11 and 12 in your submission contain map images which may be copyrighted. All PLOS content is published under the Creative Commons Attribution License (CC BY 4.0), which means that the manuscript, images, and Supporting Information files will be freely available online, and any third party is permitted to access, download, copy, distribute, and use these materials in any way, even commercially, with proper attribution. For these reasons, we cannot publish previously copyrighted maps or satellite images created using proprietary data, such as Google software (Google Maps, Street View, and Earth).

We require you to either (1) present written permission from the copyright holder to publish these figures specifically under the CC BY 4.0 license, or (2) remove the figures from your submission:

You may seek permission from the original copyright holder of Figures to publish the content specifically under the CC BY 4.0 license.

**Response:** We appreciate your concern to assure the ethical issues. However, all the aforementioned figures(2, 4, 5, 6, 7, 8, 9, 10, 11, and 12) in our manuscript are not copyrighted rather they are the result of spatial analysis that we have run in ArcGIS and SaTScan software. The GPS and DHS data that contain Shapefile and other relevant variables were obtained from the DHS office by explaining the objective of the study through online requests. Then, in order to get those figures, we import the relevant data extracted from the 2016 Ethiopian Demographic Health Survey reports and the shapefile of Ethiopia obtained from the 2016 Ethiopian Central Statistical Agency (CSA).To indicate this, we already cited the source of the shapefile alongside each figure. The shape file that we used to construct the figures can be accessed by one of the following links:

1. <https://data.humdata.org/dataset/cb58fa1f-687d-4cac-81a7-655ab1efb2d0>
2. <https://gadm.org/download_country.html>

Therefore, the maps presented in our study are not copyrighted rather they were the outputs of our spatial analysis results which are the result of those Shapefiles and projected CVS files in ArcGIS. This is the actual procedure that we employed in our present and earlier studies, as well as other Ethiopian researchers. Again we assure you that the figures presented in our study are not copyrighted but rather our spatial analysis results.

**Authors’ Response to Reviewer**#**1**

**General Comment:** If the authors address all the comments and suggestions provided, the paper has the potential to become significant for publication and beneficial for readers. However, if the recommendations are not adequately addressed, it may be advisable to decline the paper. Ensuring that all comments are thoroughly incorporated can significantly enhance the paper's quality, relevance, and contribution to the field. Therefore, it is essential for the authors to carefully consider and implement the suggested revisions to maximize the paper's impact and value for both the scientific community and readers.

**Response:** Thank you for giving us your valuable time to review the manuscript and all your thoughtful comments and suggestions. We got all of them valuable in the way to improve the paper. In the following section, you can get all our responses by highlighting them in the revised version of the manuscript.

**Comment 1:** I would like to emphasize the need for a clear explanation from the authors regarding the rationale behind conducting both multilevel and geographically weighted regression analyses. Specifically, it is essential to address why the geographically weighted regression (GWR) was chosen and how it demonstrates local statistical significance spatially. If GWR already indicates spatial significance, the inclusion of multilevel logistic regression requires justification to avoid redundancy and ensure the efficiency of the analysis. Clarification on this aspect is strongly warranted to strengthen the paper's coherence and contribute to a better understanding of the analytical approach

**Response:** Thank you for your insightful inquiry. Generally, Multilevel analysis and geographically weighted regression (GWR) are two statistical techniques that can be used to examine hierarchical and spatial data, and combining them can offer a more comprehensive understanding of complex spatial relationships. Here's why the integration of multilevel analysis as a support for geographically weighted regression is important:

1. GWR is designed to capture spatial variations in relationships between variables. By incorporating multilevel analysis, we can better account for the nested structure of the data and understand how spatial heterogeneity operates at different levels. It minimizes the prediction variance by giving more weight to nearby sample points with similar values. This helps in obtaining more reliable predictions, especially in areas where the data is sparse.
2. Combining these techniques may lead to improved model performance by accounting for both global and local variations. It helps in avoiding oversimplification of spatial relationships, offering a more nuanced and accurate representation of the data. This can have practical implications for policymakers, as it helps identify areas where interventions may be more effective or where different policies may be needed.

Regarding the second question, why the geographically weighted regression (GWR) was chosen, and how did it demonstrate local statistical significance spatially?

As there are two ways of spatial regression, namely Ordinary least square(Global model) and GWR(local model). GWR is crucial to identifying spatial predictors and that is why we used it. For more clarity we have tried to incorporate the procedures that we followed during spatial regression in the ‘spatial analysis’ section of the "Revised Manuscript with Track Changes" Page 9, Lines 244-254.

**Response to specific comments**

**Comment 1:** The methodology section lacks depth as it fails to mention the specific software utilized for spatial analysis, such as the versions of ArcGIS and SaT Scan employed.

**Response:** we entierly agree with your point of view and we have corrected and highlighted it in the ‘Abstract’ section of the "Revised Manuscript with Track Changes" Page 2, Lines 58-61.
**Comment 2:** The Results section lacks clarification on whether the outcomes represent clustering, identification of hotspot areas, or the results of the SatScan analysis. This ambiguity obscures the interpretation of the findings and their significance. To enhance clarity and provide a comprehensive understanding, it is crucial to explicitly state the nature of the outcomes, particularly regarding clustering, hotspot identification, and the results derived from the SatScan analysis. This clarification will enable readers to better comprehend the implications of the study's findings and their relevance to the research objectives.

**Response:** we appreciate your meticulous review and we also understand it. We have tried to make the result section more clear by including other findings. The revised statements were highlighted in the ‘Abstract’ section of the "Revised Manuscript with Track Changes" Page 2, Lines 65-69.
**Comment 3:** In conclusion, it is evident that the paper lacks a comprehensive and insightful summary based on the findings presented. The absence of a conclusion section deprives readers of a clear synthesis of the research outcomes and their implications. Therefore, it is imperative to craft a conclusion that effectively encapsulates the key findings, discusses their significance, and provides insights for future research or practical applications

**Response:** Again, we reconsidered it as per your suggestion and we corrected it. The corrected statements were highlighted in the ‘Abstract’ section of the "Revised Manuscript with Track Changes" Page 2, Lines 77-81.
**Comment 4:** The paper overlooks an essential aspect by failing to mention the data source and provide a direct link to the DHS portal site. Including this information is crucial for transparency and reproducibility, as it enables readers to access the original data for validation or further analysis. Therefore, it is imperative to incorporate a clear statement specifying the data source and providing a hyperlink to the DHS portal site, ensuring that readers have easy access to the dataset used in the study. This addition will enhance the paper's credibility and facilitate future research endeavors by enabling others to replicate or build upon the findings.

**Response:** thank you for your comment and suggestion. We admit that we ignored the main part and now we incorporated that information in the ‘Study area, period and data source’ section of the "Revised Manuscript with Track Changes" Page 4, Lines 142-144.

**Comment 5:** The absence of a map depicting the study area is a notable omission in the paper. Including a map provides essential context for readers to visualize the geographical scope of the study and understand the spatial distribution of the variables under investigation. Incorporating a map showcasing the study area not only enhances the clarity of the research but also enables readers to better interpret the findings in relation to geographic features and boundaries. Therefore, it is recommended to include a detailed map illustrating the study area, its relevant geographical features, and any pertinent spatial data layers. This addition will enrich the paper by providing a visual representation of the research context, thereby enhancing its overall comprehensibility and impact.

**Response:** thank you for your valuable comment. Accordingly, we have added the administrative map of the country as per your suggestion in Figure 1 and highlighted it in the ‘Study area, period and data source’ section of the "Revised Manuscript with Track Changes" Page 5, Line 148.

**Comment 6:** The author's focus solely on community-level variables limited to region and residence overlooks other potentially significant factors such as community-level educational status, wealth status, media exposure, and others. These variables play crucial roles in shaping community dynamics and could significantly influence the outcomes under investigation. Therefore, it is essential to address this oversight and consider incorporating a broader range of community-level variables in the analysis. By including additional variables such as educational status, wealth status, and media exposure at the community level, the study can provide a more comprehensive understanding of the factors impacting the outcomes of interest. This expansion of variables will enrich the analysis and enhance the depth of insights derived from the research findings.

**Response:** we appreciate your meticulous review and insightful suggestions, Accordingly, we included all the above-mentioned community-level factors and highlighted them in Tables 1,2, and 3 of the "Revised Manuscript with Track Changes’’

**Comment 7:** The absence of incremental autocorrelation analysis in the data analysis section is a notable oversight, particularly concerning autocorrelation analysis. Incremental autocorrelation analysis is crucial for understanding spatial dependence at varying distance thresholds, which can reveal important insights into the clustering patterns within the dataset. Without considering distance thresholds, the autocorrelation analysis may lack precision and potentially overlook significant spatial clustering trends. Therefore, it is imperative to address this deficiency and incorporate incremental autocorrelation analysis into the data analysis methodology

**Response:** Thank you for your insightful comment and suggestion. Accordingly, we incorporated and highlighted it in the ‘Spatial and incremental autocorrelation’ section of the "Revised Manuscript with Track Changes" Page 8, Lines 209-215. We also reported the findings in the ‘Results’ section, Page 14, Lines 325-329. We added the details as supportive information (S1File)

**Comment 8:** The Median Odds Ratio (MOR) in the Results section of the multilevel mixed-effect logistic regression analysis is not mentioned, especially considering its importance in understanding the variation between areas of highest and lowest risk. MOR provides valuable insights into the heterogeneity between different geographical areas, highlighting the extent to which individual-level characteristics versus contextual factors contribute to the observed variations in risk. Therefore, it is crucial to address this omission and include MOR analysis in the Results.
**Response:** We appreciate your meticulous review and insightful comment. As per your suggestion, we have added both the narration and the results of MOR and highlighted them in the ‘method’ (Page 10, Lines 275-277) and Result (Page 17, Lines 423-429) of the "Revised Manuscript with Track Changes".

**Comment 9:** The discussion section appears to lack depth in its interpretation of the findings, particularly in integrating and comparing results from different analytical approaches such as hotspot analysis, SatScan, and others. Each of these methods provides unique insights into spatial patterns and clusters, and their integration can enrich the understanding of the phenomenon under study. Therefore, it is essential to thoroughly discuss and contrast the findings obtained from each method, providing justification for any discrepancies observed

**Response:** Thank you for your indepth review of the manuscript and suggesting us to strengthen the discussion section. Accordingly we have made some amendments and highlighted them throughout the discussion section of the "Revised Manuscript with Track Changes", respectively. To compare and contrast our findings with other studies conducted elsewhere, we encountered a dearth of similar studies on the subject, and we hope you value our concern.

Thank you for your constructive comments and suggestions, which we got as valuable input in the improvement of the manuscript. We received all of them as a valuable contribution to our ongoing work.

**_____________________________END________________________________________**

**THANK YOU!!!**

**Authors’ Response to Reviewer**#**2**

**Comment 1:** The abstract section you provided appears to be generally well-written and provides a clear overview of the study's objectives, methods, and findings. However, there are a few areas where the abstract could be improved:

**Response:** thank you for your constructive feedback. In the following sections, we tried to address all your suggestions to improve the abstract.

**Comment 2:** Background: It would be helpful to include more detailed information in the background section regarding the research that have been done on Ethiopian women's awareness of HIV/AIDS. It would be useful to specify the number of studies, their scope, and any important conclusions or limits rather than just saying that "some studies" were undertaken. If your goal is to investigate the gaps in your knowledge, then please be content with that.

**Response:** Thank you for your meticulous review and valuable suggestion. Accordingly, we tried to make some amendments to the statement and highlighted it in the ‘Abstract’ section of the "Revised Manuscript with Track Changes", Page 2, Lines 48-50. As the abstract section needs to be short and precise, we merely focus on notifying the actual gaps of previously conducted studies and the readers can access the details of the gaps in the ‘introduction’ section.

**Comment 3:** Objective: Where are the predictors found by the regression analysis in this case that the author stated, "Consequently, this study attempted to assess the prevalence, geographical variation (Hotspots), spatial predictors, and multilevel correlations of inadequate HIV/AIDS awareness among Ethiopian women"? Because of this, the spatial regression method was used to identify your predictors instead of the multilevel regression analysis. What does little understanding of HIV/AIDS mean in terms of multilayer correlates? The writers must choose the appropriate regression model and have a well-defined target.

**Response:** Thank you for your insightful inquiry. As a geographically weighted regression (GWR) is more specific in identifying possible predictors, we also used a multilevel logistic regression to widen our chance of identifying significant predictors. On the way, we also got variables that were not significant in a GWR but in multilevel analysis. in addition, most currently conducted studies were done in this way by assuming combining these techniques may lead to improved model performance and this can have practical implications for policymakers, as it helps identify areas where interventions may be more effective or where different policies may be needed.

**Comment 4:** Methods: Although the study's use of data from the 2016 Ethiopian Demographic and Health Survey is noted, it would be beneficial to include a brief overview of the survey's methodology and sampling plan. This would provide readers with a clearer picture of the sample's representativeness and the source of the data.

**Response:** We appreciate your insightful suggestion and we made slight corrections in the way that can address your concerns. The added statement was highlighted in the ‘Abstract’ section of the "Revised Manuscript with Track Changes", Page2, Lines 55-57. To keep the simplicity of the abstract, the details were mentioned in the method section of the "Revised Manuscript with Track Changes"

**Comment 5:** Results: The prevalence of inadequate knowledge about HIV/AIDS among Ethiopian women is presented in the abstract; nonetheless, it would be helpful to give a brief overview of the main multilevel and spatial correlates of inadequate knowledge. This would help readers understand the causes of insufficient knowledge and the extent of their impacts.

Which source did you use to get the reference group variables?

**Response:** thank you for your suggestion that we agreed upon. We tried to report additional reports and highlighted them in the ‘Abstract’ section of the "Revised Manuscript with Track Changes", Page 2, Lines 65-69. Regarding the reference category of each variable, it was determined based on previous studies, and the details were incorporated in the ‘result’ section of the manuscript. We really appreciate your suggestion to incorporate all the details, however, it might be difficult for us to incorporate every detail in the abstract section due to a word limit and to make it more short and precise. We kindly suggest you and the readers to look into the document, for more detail.

**Comment 6:** Conclusion: Expanding the conclusion section to include more detailed suggestions based on the data would be beneficial. For instance, it might outline the precise treatments or tactics that could be used to close the observed knowledge gaps, rather than just recommending the need for customized health education and awareness campaigns.

**Response:** Thank you for your insightful suggestion. Accordingly, we tried to make a conclusion and recommendation based on our findings and we also incorporated those possible interventions and strategies to narrow the knowledge gap. The amended paragraph is highlighted in the ‘Abstract’ section of the "Revised Manuscript with Track Changes", Page 2, Lines 79-81.

**Comment 7:** Structure: There is room for improvement in the introduction's structure. Think about breaking up the section into paragraphs that each focus on a different facet of the subject. The content would flow more easily for readers as a result.

**Response:** thank you for your suggestion and we tried to correct it accordingly to enhance the readability of the work.

**Comment 8:** Citations: It would be beneficial to give precise citations for each piece of material, even though you have included some references to back up the claims stated in the introduction. This will guarantee the accuracy of the material provided and allow readers the chance to go further into the research that are cited for further details.

Clarity and Conciseness: The introduction has a few long and complicated sentences that may be challenging to grasp. To make the text easier to understand, try to make the language simpler and divide longer, more complicated statements into shorter, more direct ones.

**Response:** We appreciate your meticulous review and valuable insights which are helpful to enhance the quality of our work. Both issues have to be addressed and we tried to correct them in the revised version of the manuscript. All the corrected and simplified statements were highlighted throughout the ‘introduction’ section of the "Revised Manuscript with Track Changes"

**Comment 9:** Research Gap: Although the introduction notes that earlier research on Ethiopian women's comprehensive knowledge of HIV/AIDS was small in scale and scope, it would be helpful to highlight the particular research gap that this study attempts to fill. Indicate exactly how this study is different from the others and what special contributions it will make. In comparison to other earlier studies, the research gap was not often addressed or stated very clearly.

**Response:** we entirely agree with your suggestion, and accordingly we have tried to show the clear gaps in the previous studies which were addressed by the current one. The added statement was highlighted in the ‘introduction’ section of the "Revised Manuscript with Track Changes", Page 4, Lines 120-126

**Comment 10:** Significance: The study's possible ramifications for community empowerment, policy decisions, and focused interventions are touched upon in passing in the introduction. Explain the wider significance of filling the identified research gap as well as any possible practical ramifications in order to strengthen this section.

Response: thank you for your suggestion. We have incorporated and highlighted it in the ‘introduction’ section of the "Revised Manuscript with Track Changes", Page 4, Lines 135-139

**Comment 11:** Regarding SDG 3, the references 3 and 4 were incorrect. Kindly make use of the primary source for your information.

**Response:** we have corrected and highlighted it on Page 2, Line 99 of the "Revised Manuscript with Track Changes"

**Comment 12:** Some of the references are outdated (e.g. reference 13 was published in 1997)

**Response:** thank you for your meticulous review. We also got this specific reference irrelevant and we replaced it with a more relevant and updated one.

**Comment 13:** There are instances of misspellings, acronyms, and capitalizations throughout the entire document. Therefore, kindly make the necessary revisions.

**Response:** thank you for your comment. We give due emphasis to those issues and we have corrected them throughout the revised version of the manuscript.

**Comment 14:** First create an independent section about the study setting, and period, before you go to the data and population section.

**Response:** we appreciate your valuable comment which was also raised by another reviewer. We have corrected it in the revised version of the manuscript and we kindly ask you to check over it on Page 5 of the "Revised Manuscript with Track Changes’’

**Comment 15:** Women who have missing data about their residential area locations via the geographical positioning system (GPS) were excluded from the spatial analysis. How did you do it? Does this meet the proper exclusion criteria for your study over the other criteria? Create an independent section titled “Source and Study Population," and then you might narrate the criteria for your study.

**Response:** Thank you for your insightful inquiry. Yes, it has a procedure to exclude those respondents with no GPS data(i.e. with 0 latitude and 0 longitude values) by merging the outcome variable with the shape file that contains the geographic coordinates, and that was what we made. We incorporate a sub-section titled ‘Population of the study and eligibility criteria’ and we tried to address your comments accordingly, Page 5

**Comment 16:** Please kindly include more depth information about it for readers. We did not expect to ready your references always. Put them according to the previous studies or the DHS information (for illustration urban versus rural household allocations, and total EAs in Ethiopia were not addressed). Furthermore, the authors did not inform us about data collection tools, however, they put it in the title as a subsection in the methods.

**Response:** thank you for your comment. As per your suggestion, we have incorporated the details of the sampling procedures and data collection section under the ‘sampling procedure and data collection’ section of the "Revised Manuscript with Track Changes’’, Page 5, Lines 158-168.

**Comment 17:** References 21, 27, and 28 are not your appropriate evidence. Moreover, the publication data (1998) and the information inside it are quite different with regard to your interest. Kindly revise them.

**Response:** Thank you for your meticulous review. We have corrected them accordingly. However some of the references that we used in the dta analysis section especially the spatial analysis part seem outdated because they are with the scientific information about the interpretation of the outputs

**Comment 18:** The author should see it again in this section. First, the outcome ascertainment should be based on the guide to DHS, not on the interest of the author. Second, utilizing “mean” on compressive knowledge is not recommended by the guidelines; however, the author did it. Why? Based on what criteria or reference they used, Third, adequate and inadequate categorization should be read again by the author. Fourth, there are also attitude- or practice-related questions in their tool; however, they treated them for knowledge, and this should be taken into consideration. Finally, the author provided a reference for their outcome classification (reference 29); however, this reference was not the right reference for the outcome of interest. Outcome ascertainment is the heart of every research project and should be corrected properly before going to the next step.

**Response:** thank you for your insightful review and inquiry. from the beginning, we tried to consider and discuss all the issues you raised before conducting this study. Regarding the outcome measurement, all the items we used were about the women’s knowledge those selected 11 items with a response category of ‘yes’ or ‘no’. we use many items to come up with more comprehensive evidence. We kindly ask you to look into Table 3 of the revised manuscript, all those items try to measure women’s knowledge in the area of interest. We tried to follow the ways that the DHS did to measure the outcome by incorporating all those items.

Regarding using the mean as a cut of point is due to the following importance.

- It simplifies analysis by providing a straightforward reference point.
- It aids in interpretation by providing a clear benchmark for comparison.
- Using the mean as a cutoff helps standardize comparisons across different studies or populations by providing a common reference point that can be used to compare outcomes across various contexts, and
- In certain practical contexts, such as clinical settings or policy decisions, using the mean as a cutoff can simplify decision-making processes.

Regarding the reference mentioned, it has a slight difference with our ways of measurement and thus, we removed it.

**Comment 19:** The author missed some important variables such as working or employment status, ANC, place of delivery, health visits, modern contraceptive utilization (the given contraceptive utilization should be clearly defined rather than using any methods), sex of the given household head, breastfeeding status, and other individual-level factors. Fortunately, all these variables are available in the EDHS 2016 dataset. Furthermore, the author failed to include important community-level factors, such as community women's wealth, community women's education level, community-level ANC coverage, community-level women's mass media exposure, and so on. Kindly include all these and other important variables in your outcome.

**Response:** we appreciate your meticulous review. Regarding variables like ANC timing and place of delivery, they contained more than 50% missing values, and thus we omitted them. The other community-level factors were added and highlighted in Tables 1, 2, and 3 of the "Revised Manuscript with Track Changes’’

**Comment 20:** The variable selection and categorization should be done in an acceptable manner. For instance, media exposure was better classified into either of the three types (TV, radio, or newspapers). Having a mobile phone or not will not have a great implication in the context of Ethiopia. (Assume your data collection period.)here

**Response:** Thank you for your suggestion. However, about 30% of the respondents owed mobile during the period of data collection, and looking into this variable might have some importance, and it also showed a positive association in the bivariable regression.

**Comment 21:** Why used weighting factor v005/1000000, why not other numbers like 1000, or 10000000

**Response:** thank you for your inquiry. As DHS data including EDHS have a hierarchical nature, sampling weights are crucial when analyzing survey data because they help to correct for any imbalances or biases in the sampling process. Here's why weighting is important when using DHS women or IR (individual recode) files with `v005`:

- DHS surveys typically use complex survey designs, including stratification, clustering, and unequal selection probabilities, weighting adjusts for these design features to ensure that the sample accurately represents the population of interest. Without weighting, the results may not generalize well to the entire population.
- To correct for unequal selection probabilities of study participants which is common in DHS. Some groups may be oversampled or undersampled relative to their actual proportion in the population andweighting adjusts for these differences.
- By applying weighting, we can produce more accurate estimates of population parameters and reduce the standard errors associated with those estimates.

In summary, weighting is essential when using DHS data of women(IR) files with the v005 variable because it ensures that survey estimates are representative, unbiased, and precise, enabling valid inference and informed decision-making, and using 10^6^ is a standard number setted by the DHS office as a weighting factor.

**Comment 22:** If you used GWR, why did you use a multilevel analysis again? or you did not do GWR. The GWR is the regression component for the spatial analysis, and if you applied the GWR, then your probability of coming back again to the multilevel analysis is not feasible. Spatial and multilevel analyses are quite different approaches, and the best regression for you is GWR rather than GLMM.

**Response:** Thank you for asking. From the beginning, we intended to assess the determinants from the individual, community, and spatial perspectives, and that was why performed both types of regressions. Doing it in such a way has some benefits:

1. GWR is designed to capture spatial variations in relationships between variables. By incorporating multilevel analysis, we can better account for the nested structure of the data and understand how spatial heterogeneity operates at different levels. It minimizes the prediction variance by giving more weight to nearby sample points with similar values. This helps in obtaining more reliable predictions, especially in areas where the data is sparse.
2. Combining these techniques may lead to improved model performance by accounting for both global and local variations. It helps in avoiding oversimplification of spatial relationships, offering a more nuanced and accurate representation of the data. This can have practical implications for policymakers, as it helps identify areas where interventions may be more effective or where different policies may be needed.

Moreover, most of the currently conducted studies using DHS data used such ways of data analysis.

**Comment 23:** Use the phrase multilevel or mixed effect analysis separately, they are the same thing. Don’t use them as multilevel mixed-effect analysis

**Response:** Thank you for your suggestion. We have corrected it accordingly throughout the revised version of the manuscript.

**Comment 24:** The outcome variable categorization should be the same for the spatial and regression analyses, either inadequate or adequate knowledge about HIV/AIDS

**Response:** yes, of course. We already have done that way in both cases by focusing on factors associated with inadequate HIV/AIDS comprehensive Knowledge.

**Comment 25**: Use either AIC, or BIC. These two are have different assumptions and working correlation regarding the complexity and parameters of the given model

**Response**: we accept your comment and we only reported one of them;AIC

**Comment 26**: Although the data has missing values, the author failed to inform how they managed the missing values.

**Response**: Thank you for asking to maintain a proper estimation we make a great caution for the inclusion of varaibles with missing values. Some of the above-mentioned variables (ANC, place of delivery) had more than 50% missing values and we removed them from the analysis. However, we assured you that all the variables in the model were free of missing values.

**Comment 27**: Somalia Vs Somali, should be corrected

**Response**: the correct one is Somali and we make it consistent throughout the revised version of the manuscript.

**Comment 28**: Put the number before the percentage. For instance, (876 (34.3%)

**Response**: we did accordingly

**Comment 29**: There is inconsistency of variable utilization throughout the manuscript. Variables from the regression table should be incorporated in the descriptive sections first

**Response**: as per your suggestion, we have checked the presence of all variables in the descriptive part prior to proceeding to the regression model.

**Comment 30**: Put the PCV, and MOR

**Response**: we included both values in Table 6 of the "Revised Manuscript with Track Changes’’, Page 18

**Comment 31**: Please interpret and talk about the OLS model Diagnosis in your article

**Response**: we did accordingly, and we kindly suggest you look into Page 9 of the ‘Method’ and Page 14 of the ‘Results’ sections under the ‘Spatial regression’ section of the "Revised Manuscript with Track Changes’’

**Comment 32**: Use either AOR or aOR, including the abstract section

**Response**: we used AOR throughout the revised manuscript the one in the abstract was corrected and highlighted, Line 62, Page 2

**Comment 33**: The discussion primarily presents the findings without delving into their underlying causes or providing in-depth interpretations. For example, when discussing the determinants of inadequate comprehensive knowledge, it would be beneficial to explore the mechanisms through which factors such as education, wealth quintile, autonomy, and media exposure influence knowledge levels. Providing a more nuanced understanding of these relationships would enhance the discussion.

**Response**: We appreciate your insightful suggestion and we have made some modifications in the discussion section by giving due emphasis for your comment. The amended versions were highlighted throughout the ‘discussion’ section of the "Revised Manuscript with Track Changes’’

**Comment 34**: Comparison with Other Studies: The discussion includes comparisons with studies conducted in other countries, highlighting differences in knowledge levels. However, it would be helpful to provide more context by discussing potential reasons for the variations observed. What are the specific factors that contribute to higher or lower knowledge levels in different countries? This would allow for a more meaningful comparison and a deeper understanding of the factors influencing knowledge levels.

**Response**: thank you for your suggestion. We also give due emphasis to the comparison of the magnitude across different studies with possible justification for the differences. The amended versions were highlighted throughout the ‘discussion’ section of the "Revised Manuscript with Track Changes’’

**Comment 35**: Implications and Recommendations: While the discussion briefly mentions the need for tailored interventions and policy initiatives, it could benefit from more concrete recommendations based on the study's findings. For instance, how can public health campaigns, education programs, and policy initiatives be designed to address the identified determinants of inadequate knowledge? What specific strategies can be employed to improve access to education, healthcare, and media exposure? Providing practical recommendations would enhance the applicability of the study's findings.

**Response**: Thank you for giving us a crucial point of view. We reconsidered it and now we tried to make our recommendation more practical, and in line with our findings. The amended version is highlighted both in the ‘abstract’ and ‘conclusion’ section of the "Revised Manuscript with Track Changes’’.

**Comment 36**: The discussion mentions the limitations of the study, such as the cross-sectional nature of the data and the potential for recall and social desirability biases. However, it would be beneficial to discuss these limitations in more detail and consider their impact on the study's findings. Additionally, it would be helpful to suggest avenues for future research that could address these limitations and provide a more robust understanding of HIV/AIDS knowledge among Ethiopian women.

**Response**: we totally accept your suggestion, and we corrected it accordingly. The amended statements were highlighted in the ‘Discussion’ section of the "Revised Manuscript with Track Changes’’, Lines 546-551, Page 23.

Thank you for your constructive comments and suggestions, which we got as valuable input in the improvement of the manuscript. We received all of them as a valuable contribution to our ongoing work.

**_____________________________END________________________________________**

**THANK YOU!!!**

**Authors’ response to Reviewer #3**

**General comment:** This is very interesting paper as it incorporates different statistical approaches to identify factors associated with inadequate HIV/AIDS knowledge among Ethiopian women

**Response:** Thank you for giving us your valuable time to review our work and for your constructive comments and suggestions, which we got as valuable input in the improvement of the manuscript.

**Comment 1:** The prevalence of inadequate HIV/AIDS knowledge among Ethiopian women was reported 48.9%. Considering the cross-sectional nature of the data and the large prevalence of the outcome variable, odds ratio might overestimate the association between the dependent and independent variables. Therefore in my view multilevel Poisson regression analysis with robust variance would be preferable.

**Response:** Thank you for your insightful inquiry. Of course, as the outcome could be considered as a count variable, and using a Generalized linear model might be preferable (Poisson, or binomial regressions, based on their assumption). However, we thought that it would be better to look into the influence predictors at spatial and multilevel on same category of outcome variable (i.e. inadequate vs adequate knowledge) as a dichotomous variable.

**Comment 2:** To show the scale of spatial variation in the relationship between the outcome and predictors, the GWR bandwidth (whether adaptive or fixed) should be reported.

**Response**: thank you for your insightful inquiry. As per your suggestion, we reported the adaptive bandwidth of each predictor accordingly and highlighted them in the ‘results’ section of the "Revised Manuscript with Track Changes’’, Pages 16 and 17 under the reports of GWR.

**Comment 3:** In the Satscan analysis, the map with circular windows should be mapped

**Response:** We entirely agree with your suggestion. However, all the SaTScan analysis results were presented as a supportive file S2File to avoid redundancy. Thus, we kindly suggest you look into it

**Comment 4:** In the interpretation of figures 10, 11, and 12, negative coefficients need to be interpreted. The authors should also consider demonstrating what positive or negative coefficients mean.

**Response:** The sign of a coefficient indicates the direction and magnitude of the relationship between the predictor variable and the response variable. If a coefficient in GWR is negative, it suggests that there is a negative relationship between the predictor variable and the response variable at that specific location. In other words, as the value of the predictor variable increases, the value of the response variable decreases, and vice versa. We have highlighted the interpretation in the ‘results’ section of the "Revised Manuscript with Track Changes’’, Lines 410-412, Page 17.

**Comment 5:** The GWR analysis, which is the major part of the study was not discussed. The authors should discuss the findings well.

**Response:** Thank you for your meticulous review. The majority of geographical predictors in GWR showed significant associations with the outcome of interest in a multilevel logistic regression. This is why we discussed concurrently to prevent redundancy. However, we can assure you that we did not forget any of the variables without looking at them.

Thank you for your constructive comments and suggestions, which we got as valuable input in the improvement of the manuscript. We received all of them as a valuable contribution to our ongoing work.

**_____________________________END________________________________________**

**THANK YOU!!!**

**Authors’ response to Reviewer #4**

**General comment:** I would like to say thank you for considering me to review your paper which is current issue entitled as “Spatial variation and predictors of inadequate HIV/AIDS knowledge among Ethiopian Women: A spatial and multilevel analyses of the 2016 Demographic Health Survey”.

Generally, it is interesting title, information is current issue in the world and the information is so far limited, however the data source is too outdated. Saying this the following massage and question should be addressed.

**Response:** Thank you for giving us your valuable time to review our work and for your constructive comments and suggestions, which we got as valuable input in the improvement of the manuscript.

**Comment 1:** You should revise the manuscript based on Plose one manuscript preparation guideline

**Response:** Thank you for your reminder. We strictly followed the journal guidlenes from the beginning.

**Comment 2:** In your manuscript, you have used abbreviation in title and abstract part. Do you believe this is appropriate and advisable in scientific research journal?

**Response:** thank you for your inquiry. As HIV/AIDS is an acronym known by the majority of the scientific community it would not be that much problematic to write in that form as a great majority of articles did.

**Comment 3:** Should write the study design clearly you used from your data source?

**Response:** thank you for your suggestion. We corrected it accordingly in the ‘Method’ section of the "Revised Manuscript with Track Changes’’, Line 142, Page 5.

**Comment 4:** What is the importance of doing geographically weighted regression and logistic regression simultaneously? I think geographically weighted regression is strong enough to generate your evidence?

**Response:** Thank you for asking. From the beginning, we intended to assess the determinants from the individual, community, and spatial perspectives, and that was why performed both types of regressions. Doing it in such a way has some benefits:

1. GWR is designed to capture spatial variations in relationships between variables. By incorporating multilevel analysis, we can better account for the nested structure of the data and understand how spatial heterogeneity operates at different levels. It minimizes the prediction variance by giving more weight to nearby sample points with similar values. This helps in obtaining more reliable predictions, especially in areas where the data is sparse.
2. Combining these techniques may lead to improved model performance by accounting for both global and local variations. It helps in avoiding oversimplification of spatial relationships, offering a more nuanced and accurate representation of the data. This can have practical implications for policymakers, as it helps identify areas where interventions may be more effective or where different policies may be needed.

Moreover, most of the currently conducted studies using DHS data used such ways of data analysis.

Thank you for your constructive comments and suggestions, which we got as valuable input in the improvement of our manuscript. We received all of them as a valuable contribution to our ongoing work.

**______________________________END_______________________________________**

**THANK YOU!!!**
