# Supplementary material for: Spatial variation and predictors of composite index of HIV/AIDS knowledge, attitude and behaviours among Ethiopian women: A spatial and multilevel analyses of the 2016 Demographic Health Survey
Source: PLoS One. 2024 Jun 4;19(6):e0304982. doi: 10.1371/journal.pone.0304982 (PMC11149886; doi:10.1371/journal.pone.0304982)
Supplement: Attachment — Submitted filename: Response to reviewers.docx [file pone.0304982.s008.docx]

**A point-by-point response to editor and reviewers**

**Authors’ Response to Academic Editor**

**Dear: Clement Ameh Yaro, Ph.D, Academic Editor, Plos One**

We thank you for a thorough reading and constructive comments and suggestions on our manuscript and for the opportunity to revise and resubmit. We are pleased to submit the revised version of the manuscript titled “**Spatial variation and predictors of comprehensive HIV/AIDS knowledge, attitude and behaviours among Ethiopian Women: A spatial and multilevel analyses of the 2016 Demographic Health Survey**” for your consideration in the special collection of **Plos One**. The comments of the editors and the reviewers were highly insightful and enabled us to greatly improve the quality of our manuscript. In this revised manuscript we made substantial changes to address your concerns in a point-by-point response. We appreciate your time and look forward to your response and we are very keen to incorporate further comments, if any, for the betterment of the final manuscript.

On the following pages, you will find our responses to the comments and suggestions raised by the esteemed reviewer.

Sincerely,

Aklilu Habte (MPH), corresponding author

[**aklilihabte57@gmail.com**](mailto:aklilihabte57@gmail.com)

**Authors’ Response to Reviewer**#**2**

**Comment 1:** I beg you to disagree about your outcome ascertainment technique. I have seen the guidelines for DHS statistics, and they do not support your methods of outcome ascertainment.

**Response:** we appreciate your insightful comment and allowing us to look into the outcome assessment in detail, and we entirely agree with your point of view that the way of HIV/AIDS knowledge assessment and the way we did show slight variation. Accordingly, we have changed the name of the outcome variable from merely comprehensive knowledge to comprehensive HIV/AIDS knowledge, attitude, and behaviour (HIV/AIDS-KAB). We have highlighted the correction throughout the revised version of the manuscript.

We kindly ask you to look into the revised version of the manuscript.

Thank you for your constructive comments and suggestions, which we got as valuable input in the improvement of the manuscript. We received all of them as a valuable contribution to our ongoing work.

**_____________________________END________________________________________**

**THANK YOU!!!**

**Authors’ response to Reviewer #4**

**General comment:** I have no concern about the issue I raised before. So I believe it can considered for publication

**Response:** Thank you for your positive feedback by verifying that we responded to all of your comments and providing your delightful suggestion to the editorial office for the publication of our work.

**_____________________________END________________________________________**

**THANK YOU!!!**
