# Supplementary material for: Spatial variation and predictors of composite index of HIV/AIDS knowledge, attitude and behaviours among Ethiopian women: A spatial and multilevel analyses of the 2016 Demographic Health Survey
Source: PLoS One. 2024 Jun 4;19(6):e0304982. doi: 10.1371/journal.pone.0304982 (PMC11149886; doi:10.1371/journal.pone.0304982)
Supplement: Attachment — Submitted filename: Review for HIV time.docx [file pone.0304982.s009.docx]

Review to the authors

**Title changed**

It is important to ensure consistency between the title of a research article and its content to accurately represent the study's focus and findings. In this case, it seems that the title was changed to incorporate the broader scope of the study, including not only HIV/AIDS knowledge but also attitudes and behaviors among Ethiopian women. However, if the content of the manuscript remains unchanged, including the spatial analysis, outcome variable, sample size, and other aspects, it may create confusion or misalignment between the title and the actual study. This could potentially lead to discrepancies or misunderstandings among readers. In this case the author changed their title without any justification. Please see previous published researches. If you your title has been published with similar outcome, model, and data. You can access them online. At this stage follow the guideline at least. I will not review this again, if you do not follow the guideline.

To address this issue, it is recommended that the authors carefully review the manuscript to ensure that any changes made in the title are appropriately reflected throughout the article. This includes updating relevant sections, such as the introduction, methods, and discussion, to align with the expanded scope of the study as reflected in the new title. Additionally, the authors may want to consider providing a brief explanation or justification within the manuscript, clarifying the reasons for the title change and emphasizing that the content of the study remains unchanged. This can help readers better understand the study's objectives and avoid any confusion arising from the inconsistency between the title and the manuscript's content.

**Outcome variable**

I repeatedly mentioned to you that the way the authors determined the outcome variable is not based on the guidelines; rather, it was generated based on the authors' own assumptions. Unless the authors used the DHS questionnaires for their study, they should also adhere to the methodology outlined in the guidelines for generating the outcome variable.

For your reference, I have attached the instructions from the guidelines to help simplify the process for you.

**Comprehensive knowledge about HIV**

Percentage of women and men who know that a healthy looking person can have HIV and reject local misconceptions about transmission or prevention of HIV

Definition

1) Percentage of women and men age 15-49 who know that a healthy looking person can have HIV.

2) Percentage of women and men age 15-49 who know that HIV cannot be transmitted by mosquito bites.

3) Percentage of women and men age 15-49 who know that HIV cannot be transmitted by supernatural means.

4) Percentage of women and men age 15-49 who know that a person cannot become infected by sharing food with a person who has HIV.

5) Percentage of women and men age 15-49 who say that a healthy-looking person can have HIV and who reject the two most common local misconceptions.

Indicators 2, 3 and 4 are surveys-specific and may refer to other local misconceptions.

Coverage:

Population base: Women and men age 15-49

Time period: Current status at time of survey

Numerators:

**Number of women (or men) who indicate that they: 1) Know that a healthy-looking person can have HIV (women: v756 = 1; men: mv756 = 1)**

**2) Know that HIV cannot be transmitted by mosquito bites (women: v754jp = 0; men: mv754jp = 0)**

**3) Know that HIV cannot be transmitted by supernatural means (women: v823 = 0; men: mv823 = 0)**

**4) Know that a person cannot become infected by sharing food with a person who has HIV (women: v754wp = 0; men: mv754wp = 0)**

**5) Know that a healthy-looking person can have HIV (see Numerator 1 above) and reject the two most common local misconceptions about HIV transmission or prevention (these two most common misconceptions are footnoted in DHS 7 table 13.2). Survey specific but typically two of the following three (see *Calculation* below): a) HIV cannot be transmitted by mosquito bites (women: v754jp = 0; men: mv754jp = 0).**

**b) HIV cannot be transmitted by supernatural means (women: v823 = 0; men: mv823 = 0).**

**c) A person cannot become infected by sharing food with a person who has HIV (women: v754wp = 0; men: mv754wp = 0)**
