# Supplementary material for: Spatial variation and predictors of composite index of HIV/AIDS knowledge, attitude and behaviours among Ethiopian women: A spatial and multilevel analyses of the 2016 Demographic Health Survey
Source: PLoS One. 2024 Jun 4;19(6):e0304982. doi: 10.1371/journal.pone.0304982 (PMC11149886; doi:10.1371/journal.pone.0304982)
Supplement: Attachment — Submitted filename: Response to reviewers.docx [file pone.0304982.s010.docx]

**A point-by-point response to editor and reviewers**

**Dear: Clement Ameh Yaro, Ph.D, Academic Editor, Plos One**

We thank you for a thorough reading and constructive comments and suggestions on our manuscript and for the opportunity to revise and resubmit. We are pleased to submit the revised version of the manuscript titled “**Spatial Variation and Predictors of Composite Index of HIV/AIDS Knowledge, attitude and Behaviours among Ethiopian Women: A Spatial and Multilevel Analyses of the 2016 Demographic Health Survey**” for your consideration in the special collection of **Plos One**. The comments of the editors and the reviewers were highly insightful and enabled us to greatly improve the quality of our manuscript. In this revised manuscript we made substantial changes to address your concerns in a point-by-point response. We appreciate your time and look forward to your response and we are very keen to incorporate further comments, if any, for the betterment of the final manuscript.

On the following pages, you will find our responses to the comments and suggestions raised by the esteemed reviewer.

Sincerely,

Aklilu Habte (MPH), corresponding author

[**aklilihabte57@gmail.com**](mailto:aklilihabte57@gmail.com)

**Authors’ Response to Reviewer**#**2**

**Comment 1:** It is important to ensure consistency between the title of a research article and its content to accurately represent the study's focus and findings. In this case, it seems that the title was changed to incorporate the broader scope of the study, including not only HIV/AIDS knowledge but also attitudes and behaviors among Ethiopian women. However, if the content of the manuscript remains unchanged, including the spatial analysis, outcome variable, sample size, and other aspects, it may create confusion or misalignment between the title and the actual study. This could potentially lead to discrepancies or misunderstandings among readers. In this case the author changed their title without any justification. Please see previous published researches. If you your title has been published with similar outcome, model, and data. You can access them online. At this stage follow the guideline at least.

**Response:** We apologize for the inconvenience. We intended to measure the outcome variable more comprehensively. Of course, we understand that DHS assesses comprehensive HIV/AIDS knowledge using the five elements you listed. Because there has been previous research in this area, we attempted to measure comprehensive HIV/AIDS knowledge, attitude, and behavior by incorporating six additional parameters to keep the current study more novel. DHS also has some space to measure the KAB domain with the items we utilize. We kindly ask you to look into the following links:

1. <https://dhsprogram.com/publications/publication-fa56-further-analysis.cfm>
2. <https://dhsprogram.com/topics/HIV-Corner/hiv-kab/>

We kindly ask you to understand us we prefer to increase the number of items to make the study more comprehensive and novel ( there was a dearth of studies that measured the KAB domain in this way). We also want to assure you that we made a slight change in the title to address your concerns and we tried to shape all sections of the manuscript accordingly and we have highlighted them throughout the ‘Revised manuscript with track changes’

Based on the reasons that we mentioned, now we are eager to accept your esteemed decision on the final fate of the manuscript. We highly appreciate your time and effort in reviewing the manuscript.

Thank you for your constructive comments and suggestions, which we got as valuable input in the improvement of the manuscript. We received all of them as a valuable contribution to our ongoing work.

**_____________________________END________________________________________**

**THANK YOU!!!**
